# A mega-ethnography of eleven qualitative evidence syntheses exploring the experience of living with chronic non-malignant pain

**DOI:** 10.1186/s12874-017-0392-7

**Published:** 2017-08-01

**Authors:** Fran Toye, Kate Seers, Erin Hannink, Karen Barker

**Affiliations:** 1 Nuffield Orthopaedic Centre, Oxford University Hospitals NHS Foundation Trust, Oxford, UK; 2 Nuffield Department of Orthopaedics, Rheumatology and Musculoskeletal Sciences, University of Oxford, Oxford, UK; 3 Royal College of Nursing Research Institute, Warwick Medical School, University of Warwick, Coventry, UK

**Keywords:** Qualitative research, Qualitative evidence synthesis, Pain, Patient experience, Systematic review, Meta-ethnography

## Abstract

**Background:**

Each year over five million people develop chronic non-malignant pain and can experience healthcare as an adversarial struggle. The aims of this study were: (1) to bring together qualitative evidence syntheses that explore patients’ experience of living with chronic non-malignant pain and develop conceptual understanding of what it is like to live with chronic non-malignant pain for improved healthcare; (2) to undertake the first mega-ethnography of qualitative evidence syntheses using the methods of meta-ethnography.

**Methods:**

We used the seven stages of meta-ethnography refined for large studies. The innovation of *mega*-ethnography is to use conceptual findings from qualitative evidence syntheses as primary data. We searched 7 bibliographic databases from inception until February 2016 to identify qualitative evidence syntheses that explored patients’ experience of living with chronic non-malignant pain.

**Results:**

We identified 82 potential studies from 556 titles, screened 34 full text articles and included 11 qualitative evidence syntheses synthesising a total of 187 qualitative studies reporting more than 5000 international participants living with chronic pain. We abstracted concepts into 7 conceptual categories: (1) my life is impoverished and confined; (2) struggling against my body to be me; (3) the quest for the diagnostic ‘holy grail’; (4) lost personal credibility; (5) trying to keep up appearances; (6) need to be treated with dignity; and (7) deciding to end the quest for the grail is not easy. Each conceptual category was supported by at least 7 of the 11 qualitative evidence syntheses.

**Conclusions:**

This is the first mega-ethnography, or synthesis of qualitative evidence syntheses using the methods of meta-ethnography. Findings help us to understand that the decision to end the quest for a diagnosis can leave patients feeling vulnerable and this may contribute to the adversarial nature of the clinical encounter. This knowledge demonstrates that treating a patient with a sense that they are worthy of care and hearing their story is not an adjunct to, but integral to health care.

**Electronic supplementary material:**

The online version of this article (doi:10.1186/s12874-017-0392-7) contains supplementary material, which is available to authorized users.

## Background

The number of Qualitative Evidence Syntheses (QES) that aim to systematically search for and synthesise the findings from qualitative research is increasing. In 2011, Campbell and colleagues identified 41 syntheses [1]. Other reviews suggest that the number is much larger than this and that it is increasing dramatically [2, 3]. For example, Hannes and colleagues demonstrate that the number of qualitative syntheses in 2008 had doubled within four years [3]. QES is useful in health research because the proliferation of qualitative studies can sometimes make it difficult for stakeholders to utilise qualitative knowledge to inform practice and policy [4, 5]. Insights from several QES have contributed to a greater understanding of complex processes in healthcare. A few examples are: medicine taking [6], diabetes [7] antidepressants [8], chronic musculoskeletal pain [9] and chronic pelvic pain [10].

In some areas, there is a growing number of QES exploring the same or similar topics. The increasing number of QES [3, 11] with no clear way of identifying existing or planned QES raises the danger of research duplication. It also raises the question for stakeholders, which synthesis do I use? For example, we became aware of the growing number of syntheses, including our own, that explored the experience of living with chronic pain. Framed in a more positive light, an increase in QES might provide an opportunity for useful synthetic products for the purposes of policy and practice. These syntheses of QES would be useful in order to aggregate existing findings or, alternatively, to move our conceptual understanding forward. This reflects a distinction often made between QES that (a) aggregate, or (b) develop, conceptual understandings [2–4, 12–14]. Frost and colleagues explore the possibility of synthesising QES of diabetes [15]. They indicate that there has been a move away from interpretation and theory development in QES towards aggregative forms of synthesis. They caution reviewers not so simply produce ‘more of the same’ [15]. We aimed to explore whether a synthesis of QES had the potential to add a conceptual level that was greater than the sum of its QES parts. We felt it would be valuable to synthesise existing QES into a conceptual output to help us to more fully understand what it is like to live with chronic non-malignant pain.

Meta-ethnography is a method of QES that focuses on conceptualisation and theory development. As such, it is not necessarily recommended for decision-making contexts such as guideline or policy recommendations. Our aims were both substantive and methodological: (1) to bring together QES that explored patients’ experience of living with non-malignant chronic pain and develop conceptual understanding of what it is like to live with chronic non-malignant pain for improved healthcare; (2) to undertake the first mega-ethnography of QES using the methods of meta-ethnography. This is important as we know that many people living with chronic non-malignant pain continue to experience healthcare as an adversarial struggle [9]. The innovation of this study is to bring together eleven QES exploring patients experience of living with chronic non-malignant pain using the methods of meta-ethnography [12, 16]. To our knowledge, this is the first meta-ethnography of QES and we have coined the phrase ‘*mega*-ethnography’.

## Methods

We used the methods of meta-ethnography developed, refined and reported in a previous synthesis of patients’ experience of chronic musculoskeletal pain [9] and applied these methods to produce a *mega*-ethnography of QES.

### Stage 1: Getting started

There are various methods for synthesising qualitative research [2–4, 13, 14]. A distinction is often made between synthesis approaches: (a) that aggregate and decribe findings and (b) those that interpret findings and develop conceptual understanding [2–4, 12–14]. In practice these approaches overlap and it might be more useful to see these two approaches, not as dichotomous, but as two poles on a spectrum. Meta-ethnography is a conceptual approach with seven stages [12]: Stage one incorporates the rationale, aims and protocol development. Stage two involves the search, screening and quality appraisal. Stages three to six involve overlapping activities: reading the studies (stage 3), determining how the studies are related (stage 4), translating the studies into each other (stage 5), and synthesising the translations (stage 6). The final stage involves output and dissemination and of findings (stage 7).

### Stage 2: Deciding what is relevant

Although there have been calls to standardise the reporting of [17–19], and suggestions for appraising confidence in QES [5, 20], there are no agreed methods. We therefore aimed to include all QES that explored patients’ experience of chronic non-malignant pain. We searched seven bibliographic databases (medline, cinahl, psychinfo, embase, amed, HMIC, BNI) from inception until February 2016 to identify QES that explored patients’ experience of living with chronic non-malignant pain. We used the following search terms: (metasynthes* OR meta-synthes* OR “meta synthesis”) OR (metasummar* OR meta-summar* OR “meta summary”) OR (metastud* OR meta-stud* OR “meta study”) OR (metaethnog* OR meta-ethnog OR “meta ethnography”) OR (metanarrative OR meta-narrative OR “meta narrative”) OR “critical interpretive synthesis” OR (qualitative ADJ4 systematic*) OR (qualitative ADJ4 review) OR (qualitative ADJ4 synthes*) combined with (exp PAIN or pain.ti, ab). FT screened the titles, abstracts and full text of potential studies for relevance.

### Stage 3: Reading the studies

Once you have decided what to include in the synthesis, the next stage involves reading studies to identify concepts. We read the QES in alphabetical order. We maintained an excel database of the demographics reported in the primary qualitative studies included in each QES.

### Stage 4: Determining how studies are related to each other

**Fig. 1:**
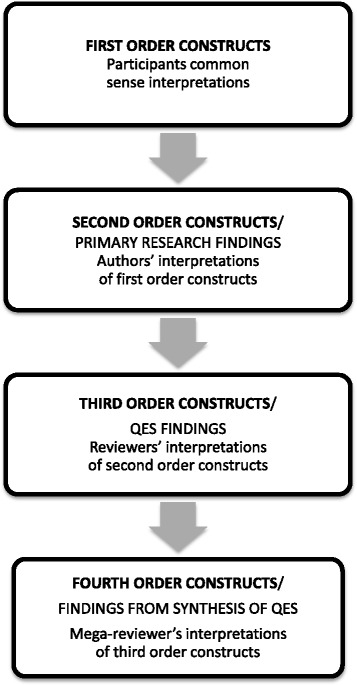
First, Second, Third and Fourth Order constructs in qualitative analysis. This figure illustrates conceptual levels in qualitative analysis: first, second, third and fourth order constructs

For meta-ethnography, *determining how studies are related* involves creating ‘a list of key metaphors, phrases, ideas and/or concepts’ [12] (page 28) from primary research studies and comparing these across studies. Schütz’ concept of *first and second order constructs* are frequently used in meta-ethnography studies to distinguish the levels of data [21]. Schütz distinguishes (1) first-order constructs (the participants’ ‘common sense’ interpretations in their own words) and (2) second order constructs (the primary researchers’ interpretations based on first order constructs). The ‘data’ of *meta*-ethnography are the primary research findings which are second order constructs. These findings are further abstracted to develop QES findings which are third order constructs (the reviewers’ interpretation of the primary authors’ interpretations). These QES findings are the data of *mega*-ethnography which are further abstracted to develop fourth order constructs (the mega-reviewers’ interpretation of reviewers’ interpretations). This is illustrated in Fig. 1. For mega-ethnography, the focus is still on conceptualising rather than describing. Two reviewers challenged each other’s interpretation of the QES findings in order to remain confident that their interpretation remained grounded in that study [16]. Any disagreements were discussed and resolved with a third reviewer. Once we had agreed upon a description of each QES finding, FT wrote a statement of this finding in the first person. For example:

*‘This theme described how their whole life and future as they knew it had been disrupted’*, becomes:

‘*My whole life and future as I know it has been disrupted’.*

We have found that writing concepts in the first person is a powerful way for reviewers and QES stakeholders to fully engage in the meaning and sentiment of each concept. It also facilitates the use of accessible language for a diverse audience in both analysis and dissemination.

### Stage 5: Translating studies into each other

The next stage involves ‘translating qualitative studies into one another’ [12]. This is done by constantly comparing concepts, observing similarities and differences and gradually organising them into abstracted conceptual categories. This process of categorisation using constant comparison is integral to thematic qualitative research methods [22]. The reviewers organised the concepts into conceptual ‘piles’ and then discussed and reorganised these piles in collaboration with each other. In other meta-ethnographies [1], researchers have used an ‘index’ paper as a way of ‘orienting the synthesis’ [23]. We did not use an index paper as the paper chosen can potentially have a dramatic effect on the resulting interpretation [24]. We read and translated each QES in alphabetical order.

### Stage 6: Synthesising translations

The next stage is to synthesise or make sense of the conceptual categories by suggesting an ‘interpretive order’ or model. Noblit and Hare originally suggested three genres of synthesis for meta-ethnography; (1) *Refutational* (where findings contradict each other), (2) *Reciprocal* (where findings are directly comparable); (3) *Line of argument* (where findings are taken together and interpreted in a theoretical model)*.* We intended to produce a line of argument by developing ‘a grounded theory that puts the similarities and differences between studies into interpretive order’ [12] (page 64). In a systematic review of methodological reporting in meta-ethnography, France and colleagues did not identify any refutational analyses [11] and raise the question ‘is refutational synthesis a necessary aspect of a good quality meta-ethnography?’ We would argue that the process of qualitative analysis is underpinned by a consideration of both reciprocity and refutation: Through constant comparison we compare similarities and differences in order to find the essence of an idea that can extend beyond its constituent parts. This resonates with the concept of idea development through a process of thesis, antithesis and synthesis in dialectic theory [25].

### Stage 7: Expressing the synthesis

The final stage involves output and dissemination of findings.

## Results

**Fig. 2:**
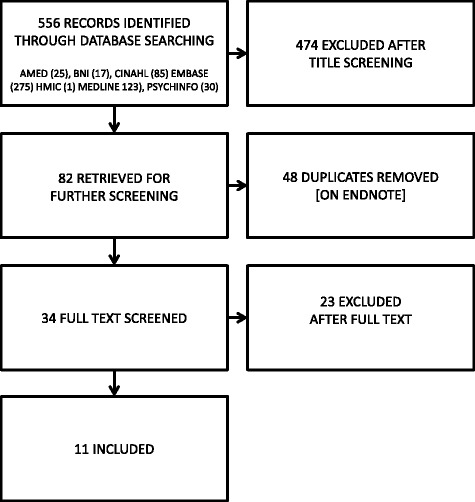
Systematic Search findings. This figure shows the records identified, screened and included in the mega-ethnography

We identified 82 potential studies from 556 titles (Fig. 2). We removed 48 duplicates and screened 34 full text articles. We excluded 23 studies for the following reasons: not specifically chronic [26–28], out of scope [29–37]; not patients experience [38, 39]; not a QES [40–45]; not possible to link QES findings to specific primary studies [46]; provided findings [47, 48] from a full report [9]. We synthesised 11 separate QES [9, 10, 49–57] exploring the experience of more than 5000 participants (*n* = 5236) living with chronic non-malignant pain in 187 published reports of 155 unique qualitative studies. It was not always clear if some of the published reports were drawn from the same sample of participants. We made the assumption that if the number and age-range of participants was identical in studies with the same first author, then findings were drawn from the same participants. Table 1 shows the author, aim of the QES, number of studies included, search date, analytic method and analytic output. One QES [10] included 21 endometriosis studies. After discussion, we included this QES as the findings from endometriosis and chronic pelvic pain had been analysed separately and the experience was underpinned by chronic pain.

**Table 1:** QES included in mega-ethnography

AUTHOR	AIM OF QES	SEARCH DATE	NUMBER OF STUDIES	ANALYTIC METHODS	ANALYTIC OUTPUT
BUNZLI ET AL. 2013 [52]	To provide a richer understanding of patients’ chronic low back pain experience.	To Oct 2011	25	Sandalowski and Barroso	8 themes
FROUD ET AL. 2014 [51]	To inform debate about outcome measure core-sets, and to suggest areas worthy of exploration within healthcare consultations for low back pain.	To July 2011	49	Meta-ethnography	5 themes with line of argument
HOPAYIAN & NOTLEY 2014 [53]	To describe the experience of health care of low back pain and sciatica patients with special reference to patients who do not receive a diagnosis.	To May 2012	28	Thematic analysis	9 themes
MACNEELA ET AL. 2015 [54]	The phenomenon of interest was the subjective experience of chronic low back pain.	To Dec 2011	38	Meta-ethnography	13 themes with line of argument
MONSIVAIS & ENGEBRETSON 2011 [56]	To examine attitudes, beliefs, behaviours, or communication issues of patients with chronic non-malignant pain or their care providers in the formal healthcare setting.	1991–2007	17	NK	4 themes
PARSONS ET AL. 2007 [55]	To review studies exploring the influence of patients’ and primary care practitioners’ beliefs and expectations on the process of care for chronic musculoskeletal pain.	To Dec 2004	15	Thematic analysis	3 themes
SIM & MADDEN 2008 [57]	To gain an interpretive understanding of the subjective impact of fibromyalgia syndrome.	To Oct 2006	23	Meta-synthesis	9 themes
SNELGROVE & LIOSSI 2013 [49]	To articulate the knowledge gained from a review of qualitative studies of patients’ experiences of chronic low back pain.	2000–2012	33	Meta-ethnography	4 themes
SOUZA ET AL. 2011 [50]	To present information regarding chronic pelvic pain and analyse the contribution of such studies to improving treatment.	1995–2010	7	NK	3 themes
TOYE ET AL. 2013 [9]	To systematically review and integrate the findings of qualitative research to increase understanding of patients’ experiences of chronic non-malignant musculoskeletal pain.	To Feb 2012	77	Meta-ethnography	11 themes with line of argument
TOYE, SEERS & BARKER 2014 [10]	To systematically review and integrate the findings of qualitative research to increase our understanding of patients’ experiences of chronic pelvic pain.	To March 2014	32	Meta-ethnography	9 themes with line of argument

**Fig. 3:**
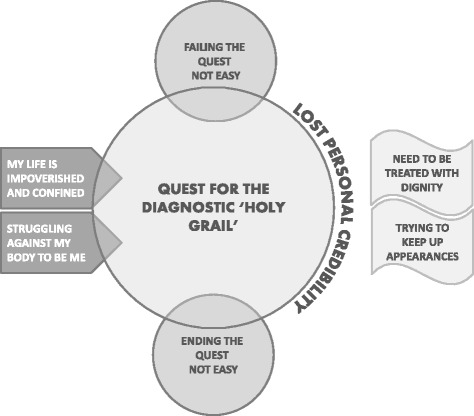
Line of argument – the imperative of the diagnostic ‘holy grail’. This figure presents our line of argument which hinges on a quest for the diagnostic ‘holy grail’. This quest is instigated by a life impoverished and confined. I am struggling against my body to be me. I take up the quest for the diagnostic ‘holy grail’ and am failing this quest. This means that I have lost personal credibility. I am therefore trying to keep up appearances and need to be treated with dignity. Deciding to end the quest for the grail is not easy

We identified 78 QES findings (Additional file 1) which did not directly refute each other and organised them into 7 conceptual categories: (1) life is impoverished and confined; (2) struggling against my body to be me; (3) the quest for the diagnostic ‘holy grail’; (4) lost personal credibility; (5) trying to keep up appearances; (6) need to be treated with dignity; and (7) deciding to end the quest for the grail. We describe each conceptual category and illustrate each with two examples of QES findings that support it. QES findings are described in the first person (Additional file 1). These descriptions are not direct quotations from the original QES. We also describe a line of argument which synthesises our conceptual categories into a whole (Fig. 3).

## My life is impoverished and confined

This conceptual category was supported by 7 out of 11 QES and describes the all-pervading nature of pain which invades all aspects of my day and night. Life is impoverished and confined. I am uncertain of what the future will bring and am confined to live in the moment.

**BUNZLI** [52]**: Psychosocial Impact of the Unpredictable, Omnipresent Nature of Pain - The nature of pain:** The pain is there day and night and disrupts everything that I do. It is unpredictable and I am always uncertain about what I can and can't do. I am dependent on my family and feel hopeless because I can do nothing in return. I am no longer able to have an intimate relationship with my partner. My role in the family and at work has changed and this can make me angry and short-tempered.

**MACNEELA** [54]**: The undermining influence of pain - Discomfort, distress, and loss:** I am in constant severe pain like someone is pulling me apart. There are good and bad days. Pain threatens every aspect of my life; I can’t sleep; I can't move; I can’t look after myself; I cannot fulfil my role; I can’t do the things I have always been able to do. My life is impoverished and confined.

## Struggling against my body to be me

This conceptual category was supported by 7 out of 11 QES and describes a struggle to maintain my sense of self. My body has become alien and malevolent and I cannot fulfil my normal duties. I am now irreparably altered.

**BUNZLI** [52]: **Psychosocial Impact of the Unpredictable, Omnipresent Nature of Pain - the changing of self:** I have lost my battle with pain to keep hold on to my personal sense of self. I am no longer what I used to be. I am no longer what I want to be. People no longer see the real me. I am ashamed of what I have become. I feel a strong sense of loss and distress. This battle to keep hold of the sense of who I am is worse than the pain.

**SNELGROVE & LIOSSI** [49]**: The impact of CLBP on self:** My pain is debilitating and I have undermined my positive and valued sense of self. It is persistent, disruptive and distressing. I have lost my previous life and I am a different person. I don’t like what I have become and I feel negative towards myself and other people. I am old before my time and have lost my dignity. I feel ashamed and helpful. I have low self-esteem and I am socially isolated. I can no longer fulfil my usual social, family and work roles. My body has become like an alien, separate from my self. Other people judge me and are unsympathetic.

## Quest for the diagnostic ‘holy grail’

This conceptual category was supported by all 11 QES and describes patients’ strong desire for a medical diagnosis. If the doctor can’t find anything then people will not believe me. I must have something or why would it hurt? I just want to find out what is wrong with me and so it can be cured.

**HOPAYIAN & NOTLEY** [53]**: Necessity of diagnosis:** A diagnosis is important to me even if there is no cure. I want to prove that there is something wrong so people believe me. I want to be certain that there is nothing serious wrong so that I can get on with managing my pain.

**SOUZA** [50]**: The importance of determining the cause of the pain:** I really need to know what is causing this pain and I will continue to look for a diagnosis. Otherwise people think it is in my head. Also they may have missed something serious. I don’t need to know the risks of this diagnostic test (laparoscopy), I just want to find out.

## Lost personal credibility

This conceptual category was supported by 10 out of 11 QES and describes a loss of personal credibility. No one believes me because there is nothing to prove that my pain is real.

**BUNZLI** [52]**: The Social Construction of CLBP - Stigmatization of CLBP:** I feel stigmatised because of my pain. The media paint a negative picture and people think that we are frauds and a burden on social welfare. Health professionals think that we are difficult or demanding or that we are just trying to get pain drugs. Employers think that we are lazy and unreliable and don’t want to give us a job. This feeling that we are bad or mad threatens my sense of self.

**TOYE** [9]**: Construct an explanation for suffering:** Pain does not fit into a medical category or diagnosis. I need a diagnosis or no one believes me. I feel worthless and ashamed. Doubt pervades my experience of pain.

## Trying to keep up appearances

This conceptual category was supported by 7 out of 11 QES and describes the need to put on a show and keep up appearances. I keep my pain to myself because I don’t want to be judged as being weak, and I don’t want to spoil things for everyone else. If I keep quiet about it no one will notice that I am no longer the person that I was.

**FROUD** [51]**: RELATIONSHIPS:** I have always been sociable person and want to join in with things. However, my relationships are suffering and I am becoming isolated. Intimate relationships have become difficult. I feel dependent on others but they are not always available. Although I need support, I tend to avoid those close to me when I am in pain. I avoid social events because I don’t want to spoil things for everybody and because I find it physically difficult to keep going. I also don’t want people to see me as I am now. If I join in with things then people won't believe that I am really in pain.

**TOYE** [9]**: Prove legitimacy:** I need to behave the right way in order to show that my pain is real. I struggle to find the right balance between looking too ill and not looking ill enough. I hide my pain so that I can appear normal but then people don’t believe me. I try and make people think that I am a good person who is not to blame for this pain.

## Need to be treated with dignity

This conceptual category was supported by 8 out of 11 QES and describes a negative experience of the healthcare system. No one is hearing my story or involving me in decisions about my care. I need to be treated with some dignity. I feel like a shuttlecock in the care system where nothing is being done to help. I feel like I am being sent around in circles.

**HOPAYIAN & NOTLEY** [53]**: Patient-practitioner relationship and interpersonal skills:** I need the clinician to listen and understand the effect that this pain is having on my life and self-image. Show an interest in me as a person and treat me as an individual.

**MONSIVAIS & ENGEBRETSON** [56]**: Specifically Requested Needs:** I expected to be told more about what is wrong with me. I expect my clinician to be caring and treat me with dignity and respect, not like I am weak or crazy. It helps me to know that they believe me and makes me feel stronger and more confident. I am frustrated to have an invisible illness and I don’t seem to be able to talk to anyone who cares.

## **Deciding to end the quest for the grail is not easy**

This conceptual category was supported by 7 QES and describes the personal challenge of giving up the quest for a diagnosis and learning to live with pain. There is a sense that this hinges upon a realisation that there is no fix for chronic pain.

**MACNEELA** [54]**: Learning to live with pain - Coming to terms with pain:** They have done all that they can for me and I have to keep going in spite of pain. I may feel better at times but there is no real cure. There is no medication that will get rid of my pain completely. I need to rely on myself. It is really difficult to come to terms with the thought of living with pain and keep a sense of purpose.

**HOPAYIAN & NOTLEY** [53]**: Outcome of care:** When I first had pain I thought that there would be a cure and now I realise I have to learn to live with it. At times I accept that all the clinicians can do is help me to cope with the situation and to reassure me. Quality of life, rather than cure, is important. At times, I cannot accept that there is no cure and think that the clinician is not committed to helping me find it. There should be cure.

## Line of argument – the imperative of the diagnostic ‘holy grail’

The final stage of meta-ethnography is to synthesise the conceptual categories into a line of argument that helps us to further understand the phenomenon of interest (Fig. 3). Through our discussions we agreed upon the imperative of the diagnostic ‘holy grail’ in people’s experience of living with chronic non-malignant pain. The quest for the diagnostic ‘holy grail’ is instigated by pain invading all aspects of a person’s life, both now and in the future (life is impoverished and confined). The person struggles against their body in order to try and keep hold of a sense of self for themselves and others (struggling against my body to be me). They thus take up a quest to find out what is wrong with their body so that it can be fixed (a quest for a diagnostic ‘holy grail’). They fall short of this quest and find nothing to prove that pain is real. People no longer believe them and they are robbed of personal credibility (lost personal credibility). They therefore try harder to keep up appearances and hide the changes so that they are not judged harshly (trying to keep up appearances). They need to be treated with dignity and to find someone in healthcare who makes them feel worthy of personal respect and who treats them as an embodied individual (need to be treated with dignity). Even if they realise that they must learn to live alongside pain in the absence of a diagnosis or cure, giving up the quest is easier said than done (deciding to end the quest for the grail is not easy).

## Discussion

Our innovation is to use the methods of meta-ethnography modified by Toye and colleagues [16] to synthesise findings from eleven QES that explore patients’ experience of living with chronic non-malignant pain. To our knowledge this is the first synthesis of QES using the methods of meta-ethnography, and we have coined the phrase, mega-ethnography. An overriding benefit of QES is that it synthesises a large body of qualitative data and makes the findings available for practise, policy and education. Our mega-ethnography synthesises concepts from eleven QES which were drawn from 187 primary qualitative studies (Additional file 2) to provide a conceptual understanding for the benefits of healthcare. The number of QES is increasing exponentially [3, 11] and whilst there is no means of identifiying existing or planned QES, duplication is likely. We felt it would be valuable to synthesise existing QES into a conceptual output to help us understand what it is like to live with chronic non-malignant pain. Meta-ethnography is a method of QES that focuses on theory development and is therefore not necessarily recommended for use in decision-making contexts such as evidence to recommendation guideline processes. More methodological testing is required to demonstrate the application of meta-ethnography in these contexts.

Some methods of QES (including meta-ethnography) abstract their individual findings into a line of argument or model. However, this is not standard amongst QES approaches. For example, some present individual findings and not a line of argument [49, 50, 52, 53, 55–57], whereas others presented a line of argument [9, 10, 51, 54]. We therefore used individual QES findings as the data for this mega-ethnography rather than their lines of argument. Both individual QES findings and lines of argument are third level constructs, although lines of argument may present a higher level of abstraction.

Our focus was on conceptual development and, although we excluded one QES where it was not possible to link QES findings to specific primary studies [46], we did not formally appraise QES quality. Although there have been calls to standardise the reporting of QES [17–19], qualitative analysis is underpinned by an interpretive framework and therefore efforts to regulate are likely to prove challenging. However, we cannot ignore the fact that some QES are indeed better (methodologically and conceptually) than others. For quantitative reviews of the effectiveness of interventions, there is now an established method for evaluating confidence in review (http://gradeworkinggroup.org/). In contrast, attempts to establish confidence in QES findings are in their infancy [5, 20]. There are two proposed method for appraising the confidence in QES which offer overlapping but distinct approaches. For QES undertaken alongside quantitative reviews of interventions, Lewin and colleagues propose four areas that allow us to determine ‘Confidence in the Evidence from Reviews of Qualitative Research (GRADE-CERQual): (1) *Methodological limitations* are the ‘extent to which there are problems in the design or conduct of the primary studies’; (2) *Relevance* is the extent to which the evidence is applicable to the QES question; (3) *Adequacy* of data is the determination of richness or weight of data supporting a finding. (4) *Coherence* considers the consistency (or inconsistency) of a particular finding across the primary studies. Munn and colleague propose two alternative but overlapping criteria for establishing ‘confidence in the output of qualitative research synthesis’ (ConQual): (1) *Dependability* aligns with the ‘methodological limitations’ of GRADE-CERQual; (2) *Credibility, is a global* evaluation of ‘fit’ between the primary data and the reviewers’ interpretations as demonstrated by adequate exemplars. This resonates with concept-indicator fit [58].

One of the challenges of attempts to determine confidence in QES is the limited agreement about what determines a ‘good’ primary qualitative study [24, 59–61]. Some feel that useful studies might be excluded from QES if our primary concern is methodological reporting rather than conceptual insight [1, 24]. It might also mean that studies embedded in other research disciplines, such as anthropology, are overlooked. Thus, a significant number of qualitative reviewers legitimately choose not to appraise for the purpose of QES [1]. This choice does not imply low quality. Indeed, although quality appraisal might help us to recognise methodological flaws, it does not necessarily help us to appraise the conceptual value of findings.

GRADE-CERQual considers *adequacy* and *coherence* of data to contribute to confidence in QES findings. Adequacy regards the vertical depth of data supporting each finding and coherence considers consistency (and difference) across the horizontal spread of data. Both constructs contribute to the gravity, or central pull of a developing idea. However, it is important to consider that gravity has a qualitative component, and that a singular idea can exert a strong gravitational pull. We should be cautious about truth-claims based purely on sheer weight or consistency of data; remember that, ‘a single word . . . can jog our memory or spark off insight where we had not expected it’ [24]. The Tale of the Emperor’s New Clothes has taught us about the validity of a small voice in the crowd. In order to demonstrate the depth and breadth of a finding, although it can be useful to provide a tally of ideas or studies supporting the finding, a single idea can stimulate a new way of thinking or highlight possible areas for further research. There is no agreed way to determine how much data provides enough gravity to support the *validity* of an idea. One idea may be *weightier* than ten other ideas. However, it is important to consider that CERQual was designed to apply to QES that are going to be used in a decision-making contexts such as a guideline panel. Further research would usefully explore the application of CERQual to conceptual QES approaches such as meta-ethnography. Although you could argue that issues of scale are not applicable to qualitative methodologies, it would be useful to consider these issues. For example, how much qualitative knowledge is adequate to support a policy decision? This issue resonates with the unresolved, even unresolvable, question ‘how many qualitative interviews is enough?’ [62]. From our experience of QES we would argue that there is confidence to be gained from performing systematic searches and incorporating a large number of ideas for comparison. However, qualitative researchers can find themselves caught between a rock and a hard place as they face criticisms for undertaking studies that are ‘too small’ (thus anecdotal) or ‘too large’ (thus not in-depth). We argue that large and in-depth studies are possible for conceptual development. Further thought should be given to the place of small idea provoking studies for policy contexts.

One of the criticisms of large QES is that it is possible to lose sight of the nuances of the primary studies. This criticism is potentially amplified in a *mega*-QES. We identified 78 QES findings that explored the experience of living with chronic non-malignant pain. At this point, the data has gone through a series of abstractions from first to fourth order construct. Some of the subtle nuances of the original QES may therefore have been lost in abstraction. For example, Toye and colleagues theme, ‘*Moving forward alongside my pain’* [9] originally incorporated several sub-themes (integrating my painful body; redefining normal; being part of a community; being open about my pain; realising that there is no cure; becoming expert). These themes became abstracted along with other QES findings into the idea, ‘deciding to end the quest for the diagnostic *holy grail’*. We did not use either of our studies [9, 10] as an index paper to ‘orient[ate] the synthesis’ [23]. Although it is unrealistic to claim that our interpretations were not influenced by our own ideas, we made every effort to keep an open mind and utilise our existing ideas as *sensitising* rather than *definitive*; ‘definitive concepts provide prescriptions of what to see, sensitising concepts merely suggest directions along which to look’ [63] (p. 7). It is useful to consider conceptual qualitative analysis as a *dialectic,* rather than *linear* process where tension between existing and new ideas can create innovative ways of thinking [26].

Although qualitative research philosophy emphasises the idiographic nature of knowledge and focuses on the unique contextual experience, we argue that it also has the power to say something valuable that is transferable beyond its context. Thus, we found that further abstraction brought conceptual gains. Importantly, the imperative of ‘the diagnostic *holy grail’* helps us to understand that investing in the healing powers of diagnosis can leave patient’s *vulnerable* [64]; if a diagnosis is not made the person in pain can suffer the consequences of lost personal credibility. This applies in situations where the person has either failed, *or* given up, the quest for a diagnosis. A decision to end the quest is therefore not taken lightly and patients may need support to make this decision. The way that we explain illness is embedded in cultural understanding and thus underpinned by powerful emotions [65].Our findings demonstrate that biomedical model is a culturally embedded model that is integral to a person’s credibility, and this may explain why it is so difficult to give up the quest for a diagnostic ‘holy grail’.

## Conclusion

The innovation of this study is to present the first mega-ethnography of QES to develop our conceptual understanding of what it is like to live in pain. Findings demonstrate that people with chronic pain are fundamentally altered by pain and often feel that health care professionals do not hear or believe them. This finding has implications for practice as lost credibility may encourage a continuing diagnostic quest. Patients may need support to make the decision to cease the continuing search for a diagnosis and cure. It would be useful for health care professionals to take affirmative action to reaffirm patients’ personal credibility through listening, hearing and believing. This part of clinical work is not an adjunct to, but integral to effective health care.

## Additional files

Additional file 1: Findings from 11 QES described in first person. (DOCX 45 kb)

Additional file 2: Primary qualitative studies included in 11 QES. (DOCX 140 kb)

## Abbreviations

ConQual: Confidence in the output of qualitative research synthesis
Evaluation: Confidence in the Evidence from Reviews of Qualitative Research
GRADE-CERQual: Grading of Recommendations Assessment Development and
NK: not known
