# Supplementary material for: A mega-ethnography of eleven qualitative evidence syntheses exploring the experience of living with chronic non-malignant pain
Source: BMC Med Res Methodol. 2017 Aug 1;17:116. doi: 10.1186/s12874-017-0392-7 (PMC5540410; doi:10.1186/s12874-017-0392-7)
Supplement: Supplementary file 1 — Findings from 11 QES described in first person. (DOCX 45 kb) [file 12874_2017_392_MOESM1_ESM.docx]

# APPENDIX 1: Findings from 11 QES described in first person

### BUNZLI 2013: The Social Construction of CLBP - Participants in the studies held biomedical beliefs about their back pain

If my doctor does not give me a medical diagnosis, no one will believe or support me at home or work. I won’t be eligible for social welfare. If I don’t have a diagnosis, how can I get better? I am really annoyed with my doctor because he is not telling me what is wrong with me. I need to find someone who will give me a diagnosis so that I know what to do. I am disenchanted with the medical system but I live in hope of a diagnosis and cure in the future.

### [BUNZLI 2013: Psychosocial Impact of the Unpredictable, Omnipresent Nature of Pain - The nature of pain](E:\\MEGA ETHNOGRAPHY\\a22ca782-5651-4b84-95d3-32ca389d3d9c)

[The pain is there day and night and disrupts everything that I do. It is unpredictable and I am always uncertain about what I can and can't do. I am dependent on my family and feel hopeless because I can do nothing in return. I am no longer able to have an intimate relationship with my partner. My role in the family and at work has changed and this can make me angry and short-tempered.](E:\\MEGA ETHNOGRAPHY\\a22ca782-5651-4b84-95d3-32ca389d3d9c)

### [BUNZLI 2013: Psychosocial Impact of the Unpredictable, Omnipresent Nature of Pain - the changing of self](E:\\MEGA ETHNOGRAPHY\\a22ca782-5651-4b84-95d3-32ca389d3d9c)

[I have lost my battle with pain to keep hold on to my personal sense of self. I am no longer what I used to be. I am no longer what I want to be. People no longer see the real me. I am ashamed of what I have become. I feel a strong sense of loss and distress. This battle to keep hold of the sense of who I am is worse than the pain.](E:\\MEGA ETHNOGRAPHY\\a22ca782-5651-4b84-95d3-32ca389d3d9c)

### [BUNZLI 2013: The Social Construction of CLBP - Stigmatization of CLBP](E:\\MEGA ETHNOGRAPHY\\a22ca782-5651-4b84-95d3-32ca389d3d9c)

[I feel stigmatised because of my pain. The media paint a negative picture and people think that we are frauds and a burden on social welfare. Health professionals think that we are difficult or demanding or that we are just trying to get pain drugs. Employers think that we are lazy and unreliable and don’t want to give us a job. This feeling that we are bad or mad threatens my sense of self.](E:\\MEGA ETHNOGRAPHY\\a22ca782-5651-4b84-95d3-32ca389d3d9c)

### [BUNZLI 2013: Coping with CLBP (Strategies to control the “assault on the self”)](E:\\MEGA ETHNOGRAPHY\\a22ca782-5651-4b84-95d3-32ca389d3d9c)

[At times I avoid social situation because I don’t want to let anyone down. I also know that there is social stigma about chronic back pain. Social withdrawal protects me from being seen as someone that I am not. However, it also means that I am isolated and this makes me feel low. I try to fight against the pain and sometimes I do too much. However, I have commitments and some things I have to do whether I have pain or not.](E:\\MEGA ETHNOGRAPHY\\a22ca782-5651-4b84-95d3-32ca389d3d9c)

### [BUNZLI 2013: Coping with CLBP - Strategies to control the omnipresent, unpredictable nature of pain](E:\\MEGA ETHNOGRAPHY\\a22ca782-5651-4b84-95d3-32ca389d3d9c)

[Pain is always present and unpredictable. I must be careful what I do and pay careful attention to what my body is telling me. I am very cautious about certain movements and avoid certain activities. Although I will take pain medication, I don’t want to become dependent pills. I also need to think about the side effects which make me feel like I am not quite myself.](E:\\MEGA ETHNOGRAPHY\\a22ca782-5651-4b84-95d3-32ca389d3d9c)

### [BUNZLI 2013: Coping with CLBP (Acceptance)](E:\\MEGA ETHNOGRAPHY\\a22ca782-5651-4b84-95d3-32ca389d3d9c)

[Despite the “battle” to control my pain and its assault on my sense of self, I know that I need to learn to live with the pain. Although acceptance can fill me with despair, it can also be a turning point to a more hopeful outlook of the future.](E:\\MEGA ETHNOGRAPHY\\a22ca782-5651-4b84-95d3-32ca389d3d9c)

### [BUNZLI 2013: The Social Construction of CLBP (Strategies to gain credibility)](E:\\MEGA ETHNOGRAPHY\\a22ca782-5651-4b84-95d3-32ca389d3d9c)

[Because others doubt my experience, I need to show that I am a trustworthy and credible person who is not to blame for having pain. I need to balance looking too ill with looking ill enough to be credible. This is very difficult because my pain fluctuates unpredictably. Sometimes I just avoid people to avoid their scrutiny. If I compare myself to other people who have pain I can see that my pain is real. Some people are the malingering type.](E:\\MEGA ETHNOGRAPHY\\a22ca782-5651-4b84-95d3-32ca389d3d9c)

### FROUD 2014: ACTIVITIES

I can’t do the things that I used to do around the house and have had to give up things that I enjoy.

### FROUD 2014: Changing outlook

I am afraid that I will lose control of my life and future. I feel overwhelmed by emotions. I want to see what is wrong on an x-ray. It makes me angry when clinicians all tell me different things, particularly if they say it is all in my head. It is confusing. It is much easier for me to move on if I have an official diagnosis. Sometimes I start to doubt the diagnosis they have given me. Since I began to realise that I might not get a diagnosis or a cure, my outlook has begun to change.

### FROUD 2014: RELATIONSHIPS

I have always been sociable person and want to join in with things. However, my relationships are suffering and I am becoming isolated. Intimate relationships have become difficult. I feel dependent on others but they are not always available. Although I need support, I tend to avoid those close to me when I am in pain. I avoid social events because I don’t want to spoil things for everybody and because I find it physically difficult to keep going. I also don’t want people to see me as I am now. If I join in with things then people won't believe that I am really in pain.

### FROUD 2014: Stigma

Family, friends, colleagues and health professionals don’t believe me because I have no proof. If I had a diagnosis people would believe me. Sometimes I even question myself. As there is no explanation for the variability and unpredictability of my pain it makes it even more difficult for people to believe me. At times I make myself look worse so that people believe me. At other times I just avoid people so that I can avoid this.

### FROUD 2014: WORK

I am worried that I will lose my job and this will make thing financially difficult. I can’t afford to take time off work or go part time. I don’t tell anyone at work about my pain because I might lose my job. At times I pretend I am taking holiday rather than admitting to being off because of pain. People will think badly of me if I keep taking time of sick. I sometimes carry on and do jobs that I shouldn’t but worry that if I can do things then people won’t believe that there is anything wrong with me. Perhaps I am no longer any good at my job.

### HOPAYIAN 2014: Necessity of diagnosis

A diagnosis is important to me even if there is no cure. I want to prove that there is something wrong so people believe me. I want to be certain that there is nothing serious wrong so that I can get on with managing my pain.

### HOPAYIAN 2014: Recognizing the expert

My GP does not have the knowledge to treat my pain. I need someone who knows what to do. Medication is not going to make me better.

### HOPAYIAN 2014: INFORMATION

I need clear and accurate information. I need the clinician to speak clearly and take time to explain things in language that I can understand. Take time to get to know me and encourage me to discuss things.

### HOPAYIAN 2014: Outcome of care

When I first had pain I thought that there would be a cure and now I realise I have to learn to live with it. At times I accept that all the clinicians can do is help me to cope with the situation and to reassure me. Quality of life, rather than cure, is important. At times, I cannot accept that there is no cure and think that the clinician is not committed to helping me find it. There should be cure.

### HOPAYIAN 2014: Personalized care

I need to be involved in the decisions about my care and my goals. I want the clinician to consider my preferences and plan my care around me as a unique individual with individual needs. I want us to make decisions about me in partnership.

### HOPAYIAN 2014: Process and content of care

Eclectic findings

I want a thorough hands-on assessment and this usually will include an x-ray or scan. I think they think it is all in my head or that I am making it up. I want to be treated as an individual.

### HOPAYIAN 2014: Patient-practitioner relationship and interpersonal skills

I need the clinician to listen and understand the effect that this pain is having on my life and self-image. Show an interest in me as a person and treat me as an individual.

### HOPAYIAN 2014: Service matters

It is really difficult to access specialists (e.g. physiotherapy). My family doctor is stopping me accessing specialist services. There are long waiting lists and it costs too much to see someone privately.

### HOPAYIAN 2014: Delegitimation

I need to feel that I have been taken seriously: seen, heard and believed. I don’t think people believe me because I look normal and this adds to my suffering. I need a diagnosis so that people believe I am telling the truth.

### MACNEELA 2015: Learning to live with pain - Attitudes to collaboration

At times I need someone to push me so that I get on with things but I don't always want to be pushed too hard or far. At times I just want to be told what to do. At times I want to take a more active role.

### MACNEELA 2015: The person as patient~ unsatisfying relationships with health care - Disappointment with health care

I am frustrated with my medical treatment. My family doctor lacks knowledge and skills in pain management. I don’t think my healthcare is individualised or personal.

### MACNEELA 2015: The undermining influence of pain - Discomfort, distress, and loss

I am in constant severe pain like someone is pulling me apart. There are good and bad days. Pain threatens every aspect of my life; I can’t sleep; I can't move; I can’t look after myself; I cannot fulfil my role; I can’t do the things I have always been able to do. My life is impoverished and confined.

### MACNEELA 2015: A disempowering impact on all levels - Family strain

Pain has affected my personal relationships. I have lost the sense of trust and mutual understanding. I feel dependent on help from those close to me and this is causing negative feelings between us even if they are sympathetic. I may lose my partner because of this. Pain is also putting a strain on the family as I can’t do the things that I used to do and that my family expect me to do.

### MACNEELA 2015: A disempowering impact on all levels - Hopelessness

Why me? Why have I got this pain? I feel weak and emotional. At times I feel angry and depressed and just want to end it all.

### MACNEELA 2015: A disempowering impact on all levels - Loss of job and lack of money

I may lose my job because of pain and will not be able to support myself or family. Work is really important to my sense of self. I can’t manage my normal role at work and I might even damage my back further at work. My work colleagues are not supportive.

### MACNEELA 2015: the person as patient~ unsatisfying relationships with health care - Listening and communication

My clinician is not listening to me. I need them to listen, even if they can’t come up with a cure. I need *someone* to listen. I need to be involved in my care decisions.

### MACNEELA 2015: The person as patient - unsatisfying relationships with health care - Needing confirmation

The label 'chronic back pain' is too vague and does not make it seem real to me or others. I need to know a specific cause or no one will believe me.

### MACNEELA 2015: Learning to live with pain - Self-management practices

I will just make adjustments and avoid the doctors. I will try and ignore my pain and continue to fight against it. Others are much worse off than me. I don’t know how long I can continue to ignore my pain. Even if I see a clinician, it is my own responsibility to manage my pain. I know it is important to do exercise and keep active but it is really difficult to stick to daily routines with pain.

### MACNEELA 2015: A disempowering impact on all levels - Social withdrawal

Back pain has a stigma attached to it and people don’t believe us. I avoid social situations for fear of being judged badly or being accused of malingering. I can’t really afford to join in with things socially anymore. I am irritable because of my pain and no one would want me around anyway.

### **MACNEELA 2015: The undermining influence of pain - Worry and fear for the future**

My future looks bleak and things may even get worse. Pain has taken over my future. I am worried that I will become dependent on others. I alternate between hope and despair.

### MACNEELA 2015: Learning to live with pain - Coming to terms with pain

They have done all that they can for me and I have to keep going in spite of pain. I may feel better at times but there is not real cure. There is not medication that will get rid of my pain completely. I need to rely on myself. It is really difficult to come to terms with the thought of living with pain and keep a sense of purpose.

### MACNEELA 2015: A disempowering impact on all levels - An oppressive intrusion on the self

Pain assaults my sense of self. It is an intruder. The good side of me is constantly struggling against the bad side. My body is unreliable. I am embarrassed about being the type to have back pain. I also feel that I am to blame in some way.

### MONSIVAIS 2011: Pain-Related Behaviours Patients May Exhibit

If clinicians are not taking my problem seriously I have to make sure that I look ill, but I worry about complaining too much. I don’t tell my clinician everything because I want to stay on good terms with them. I can’t stay in a healthcare system that does not acknowledge me or my problem.

### MONSIVAIS 2011: Beliefs and Expectations about Appropriate Treatment

I expect the clinician to give me a diagnosis, treatment and a cure for my pain. My family doctor needs to refer me on to a specialist because they are not a specialist in pain. If I do not have a diagnosis how can they treat and cure my pain?

### MONSIVAIS 2011: Appropriate Treatment Expectations Generally Not Met

A few eclectic areas

I am trapped in the health system that is not doing anything effective to help me. I don’t feel understood or believed. I need clinicians to confirm that my pain is real. I need a diagnosis so that people take me seriously. I can’t get help or benefits unless they prove that I have ‘something’ and I will need to keep going back into healthcare system in order to prove this.

### [MONSIVAIS 2011:](file:///E:\MEGA%20ETHNOGRAPHY\a187ebf8-54d4-413b-92d3-32ca3aba77ac) Specifically Requested Needs

I expected to be told more about what is wrong with me. I expect my clinician to be caring and treat me with dignity and respect, not like I am weak or crazy. It helps me to know that they believe me and makes me feel stronger and more confident. I am frustrated to have an invisible illness and I don’t seem to be able to talk to anyone who cares.

### [PARSONS 2007:](file:///E:\MEGA%20ETHNOGRAPHY\8e534b00-0a47-4d30-9bd3-32ca3b3610ca) Beliefs About Pain Causation and Influences on These Beliefs

My family doctor seems to focus on my pathology and not on me as a person

### PARSONS 2007: patient education

I am not being given clear or practical advice to follow

### PARSONS 2007: Gaining Trust through Diagnosis and Referral

I want to be believed not treated like a malingerer. A diagnosis is important to my credibility. I know the doctor is taking me seriously if he sends me for a test.

### SIM 2008: Searching for a diagnosis - Receiving a diagnosis of FMS

It was a relief when I got a diagnosis of Fibromyalgia as people believed me and I knew it wasn’t life threatening. However, I now realise that this diagnosis does not bring effective treatment or cure.

### SIM 2008: Experience of symptoms - describing pain

There are no words to express my pain to others. The pain is invisible. No one understands what I am going through. This makes it really difficult for me to cope with it.

### SIM 2008: Experience of symptoms – Fatigue

The fatigue is worse than the pain. I am constantly weary. It is impossible to maintain relationships and to do the things I have to because I am so tired.

### SIM 2008: Legitimacy

Because I look healthy people don’t believe me, particularly if I remain active. I have loads of negative test results and people question my credibility because of this. I keep my diagnosis a secret and try to appear 'normal' because of the stigma of having an illegitimate illness. Doctors lose interest in me, or don’t believe me, because I don’t have a diagnosis.

### SIM 2008: Experience of symptoms – pain

I cannot explain or get to grips with my pain. It remains ambiguous: it seems very specific at times yet hard to pin down; constant yet varying; when I am active and also when I rest; effecting mundane activities yet globally devastating; I am well yet ill; it affects my body and mind; it is worrying yet nothing to worry about; elusive yet powerfully felt; predictable yet unpredictable; Pain is not dualistic yet creates a chasm between my body and self.

### [SIM 2008: Searching for a diagnosis -](file:///E:\MEGA%20ETHNOGRAPHY\936ebc5d-b50e-4aa0-82d3-32ca3ba8817d) Post diagnosis

I tried all sorts of interventions after my diagnosis of Fibromyalgia but not relief or understanding has come. I will keep looking for something that will work.

### [SIM 2008: Searching for a diagnosis -](file:///E:\MEGA%20ETHNOGRAPHY\936ebc5d-b50e-4aa0-82d3-32ca3ba8817d) Pre-diagnosis

I have gone to so many clinicians looking for a diagnosis. What if it is life-threatening and someone has missed it? Do they think it is all in my mind if they can’t find anything? I resent being told that there is nothing wrong with me or that it is all in my head. I want a diagnosis

### SIM 2008: Experience of symptoms - Psychological problems

I am depressed, my sleep disturbed and I cannot think straight. I am losing control of what I was. I will never get or keep a job.

### SIM 2008 - COPING (RE-EVALUATION OF LIFE)

My life has really changed because of pain. I try to maintain my normal role but his has put a strain on my relationships. I can’t do all the things I used to do. I feel isolated, lonely and bereft. I have lost who I was. In some ways this has made me discover what is truly important to me. However, I try not to think about what the future holds and live one day at a time.

### [SNELGROVE 2013: Relationships with significant others (Relationships with family and friends](file:///E:\MEGA%20ETHNOGRAPHY\a4b4f897-bd2d-452f-a2d3-32ca3c210c91))

Although my family and friends can support me, it is difficult to maintain a normal family life. I cannot fulfil my role. I feel guilty and angry about this. I feel like a burden and that I am holding people back. People lose sympathy because there seems to be nothing physical wrong with me. Friends, family and health professionals are judgemental and I am not being treated fairly.

### [SNELGROVE 2013: Relationships with significant others (Health Professionals and the organisation of care)](E:\\MEGA ETHNOGRAPHY\\a4b4f897-bd2d-452f-a2d3-32ca3c210c91)

[A want a mutual partnership with my HCP where I can feel safe. I need to be recognised as an individual and get individualised care. It does not help when professionals think that I am crazy or lazy, or that I am trying to get some sort of benefit. I don’t think they believe me because they are not doing anything. I feel stigmatised and this upsets me. I try to look like I am in pain so that they believe me. I need to pass a medical test in order to get the support I need. Although I want a biomedical diagnosis, I need to know that the professional understands the personal impact of pain on my life. I am struggling to be taken seriously in health care. I continue to search for a diagnosis to support my legitimacy.](E:\\MEGA ETHNOGRAPHY\\a4b4f897-bd2d-452f-a2d3-32ca3c210c91)

### [SNELGROVE 2013:](file:///E:\MEGA%20ETHNOGRAPHY\a4b4f897-bd2d-452f-a2d3-32ca3c210c91) The impact of CLBP on self

My pain is debilitating and I have undermined my positive and valued sense of self. It is persistent, disruptive and distressing. I have lost my previous life and I am a different person. I don’t like what I have become and I feel negative towards myself and other people. I am old before my time and have lost my dignity. I feel ashamed and helpful. I have low self-esteem and I am socially isolated. I can no longer fulfil my usual social, family and work roles. My body has become like an alien, separate from my self. Other people judge me and are unsympathetic.

### SNELGROVE 2013: Coping with CLBP:

*A bit eclectic*

My pain is not going away but I am trying different strategies to try and cope with it. Staying in bed or lying down all the time is not helping. Drinking too much alcohol does not help. It is having an impact on my self-esteem. I avoid things and people so that I don’t lose face. I am becoming isolated and depressed, although I try and be stoical and appear normal.

### SOUZA 2011: Expectations regarding the doctor-patient relationship

I am frustrated with my doctor and the healthcare system. I need to know what is wrong with me. The doctor downplays my problems. They focus on the physical symptoms and not on me as a person. I find it difficult to describe my pain and I don’t understand what they are telling me. I don’t think they believe me.

### SOUZA 2011: The importance of determining the cause of the pain

I really need to know what is causing this pain and I will continue to look for a diagnosis. Otherwise people think it is in my head. Also they may have missed something serious. I don’t need to know the risks of this diagnostic test (laparoscopy), I just want to find out.

### SOUZA 2011: gender issues

If the doctor can’t find anything they think that there is nothing wrong with me, and that this is normal. This pain must be normal and I should just put up with it. It’s just my hormones and is normal for women.

### TOYE 2013: struggling to affirm self

It is a struggle to be me. My body has become alien and malevolent. I am struggling to hold onto a sense of self, although I know that I am irreparably altered. I cannot fulfil my normal roles and I feel guilty. My self-esteem is low. I don’t want to be a burden on others and therefore hide my pain. This can be a problem because if people can’t see my pain then they don’t believe me.

### TOYE 2013: Moving forward alongside my pain (Becoming an expert)

I am the expert and am becoming less reliant on healthcare. I know my body and I am confident to make my own decisions.

### TOYE 2013: Moving forward alongside my pain (Being part of a community of others)

I feel like part of a community of other people with pain, where I am valued and treated with dignity. However, I am not like all people with chronic pain.

### TOYE 2013: Construct an explanation for suffering

Pain does not fit into a medical category or diagnosis. I need a diagnosis or no one believes me. I feel worthless and ashamed. Doubt pervades my experience of pain.

### TOYE 2013: Prove legitimacy

I need to behave the right way in order to show that my pain is real. I struggle to find the right balance between looking too ill and not looking ill enough. I hide my pain so that I can appear normal but then people don’t believe me. I try and make people think that I am a good person who is not to blame for this pain.

### TOYE 2013: Moving forward alongside my pain (Listening to and integrating my painful body)

I am listening to my body and doing what it tells me to do. It is *my* body and I am working with it.

### TOYE 2013: Negotiate the healthcare system

I feel like a shuttlecock in the healthcare system as I am referred back and forth. I don’t want to keep going to the doctor because they don’t seem to do anything but I am trapped in the system. There may be some hope of a cure. I need to feel valued and treated with dignity in the system. I want a medical diagnosis but I also need to be treated like more than just a body.

### TOYE 2013: Moving forward alongside my pain (Realising that there is no cure)

I am not longer looking for a diagnosis as this has stopped me from moving on. I am gaining a new sense of who I am rather than thinking about who I used to be.

### TOYE 2013: Reconstruct self in time (construction of time altered — unpredictable now and future)

My sense of self now and in the future has been altered by pain. My pain is so unpredictable that I can only live in the moment. My life is dominated by caution and I can no longer be spontaneous. My plans and dreams are irreparably altered and my life focused inwards.

### TOYE 2013: Moving forward alongside my pain - Redefining normal

I am redefining my sense of me. I am different but still me. I can still enjoy life even thought I feel a sense of loss for my old self.

### TOYE 2013: Moving forward alongside my pain (telling others about my pain)

I know that I don’t have to hide my pain from others. I need to let people know my limitations. I don’t need anyone’s approval. I will limit the demands placed on me.

### TOYE 2014: Ambiguous experience of healthcare services

Health professionals do not validate my experience. They tell me it is just normal and that other women cope with it. They won’t refer me to a specialist. Some say it will be OK if I get pregnant. They are not hearing my story.

### TOYE 2014 Validation by diagnosis

I need a medical explanation so that I can be sure there is nothing serious and so that people believe me. I knew that there was something wrong when they finally found it (endometriosis). I am cross they took so long to find it. However, even though I have a diagnosis this doesn’t mean there is a cure.

### TOYE 2014: Embodiment of knowledge through community

I know this is real because I know my own body and there are other people like me. I am not alone with this. I have talked to other women about this shared experience.

### TOYE 2014 Elevation of experiential knowledge

I am the expert because I am the one with the experience. I am not going to do what the doctor says. My doctor doesn’t know what my experience is like.

### TOYE 2014: Social construction of ‘pathology’ vs. normal

I am struggling to know what is normal and what is pathological. They tell me it is normal. Other people just seem to get on with it.

### TOYE 2014: Relentless and overwhelming pain

My pain is horrific ad overwhelming. I am crippled by it. I am on an emotional rollercoaster. I feel angry, depressed, guilty, bereft, anxious, frustrated, afraid and full of dread.

### TOYE 2014: A culture of secrecy – managing the disclosure of pain

Everyone just thinks this is a woman’s problem. I want to keep it private so I keep it to myself and don’t tell anyone. No one talks about menstruation. I don’t know if this is just normal as no one else talks about it and I have nothing to compare with. I am isolated by my pain. Because I don’t have a diagnosis no one would believe me. It is difficult to balance the tension between hiding and showing that I have a problem. People will just think I can’t cope like other women can. I am embarrassed and ashamed and this makes me feel depressed and isolated. I don’t want people to get fed up with me. I will just put up with it.

### TOYE 2014: Threat to self

My whole life and future as I know it has been disrupted. I am struggling to lead a normal life and fulfil my expected roles. I always wanted to have a family. I am no longer me. I am not what I used to be. I used to be healthy. My body has become a threat to myself.

### TOYE 2014: Unpredictability and future

My symptoms are random and uncertain. I can’t make plans for the future. I am powerless. I might not be able to have a family
