# Supplementary material for: A mega-ethnography of eleven qualitative evidence syntheses exploring the experience of living with chronic non-malignant pain
Source: BMC Med Res Methodol. 2017 Aug 1;17:116. doi: 10.1186/s12874-017-0392-7 (PMC5540410; doi:10.1186/s12874-017-0392-7)
Supplement: Supplementary file 2 — Primary qualitative studies included in 11 QES. (DOCX 140 kb) [file 12874_2017_392_MOESM2_ESM.docx]

# Appendix 2 - Primary qualitative studies included in 11 QES

| AUTHOR & YEAR | CONDITION | COUNTRY | AGE RANGE | N | DATA COLLECTION | ANALYSIS |
| --- | --- | --- | --- | --- | --- | --- |
| AEGLER 2009[[1](#_ENREF_1)] | MSK | SWITZERLAND | 29-61 | 8 | SEMI-STRUCTURED INTERVIEW | THEMATIC ANALYSIS |
| AFRELL 2007[[2](#_ENREF_2)] | MSK | SWEDEN | 30-72 | 20 | SEMI-STRUCTURED INTERVIEW | PHENOMENOLOGY |
| ALLEGRETI 2010 [[3](#_ENREF_3)] | CBP | USA | 28-72 | 23 | SEMI-STRUCTURED INTERVIEW | THEMATIC ANALYSIS |
| ARNOLD 2008 [[4](#_ENREF_4)] | FM | USA | 31-72 | 48 | FOCUS GROUPS (FG) | GROUNDED THEORY |
| ASHBY ET.AL. 2010[[5](#_ENREF_5)] | CBP | AUSTRALIA | 23-59 | 11 | SEMI-STRUCTURED INTERVIEW & OBSERVATION | THEMATIC ANALYSIS |
| BAIR 2009 [[6](#_ENREF_6)] | MSK | USA | 27-84 | 18 | FOCUS GROUPS | THEMATIC ANALYSIS |
| BALLARD ET AL. 2006[[7](#_ENREF_7)] | ENDOMETRIOSIS | UK | 16-47 | 32 | SEMI STRUCTURED INTERVIEWS | THEMATIC, ANALYSIS |
| BALLWEG 1992[[8](#_ENREF_8)] | ENDOMETRIOSIS | USA | NK | NK | LETTERS | NK |
| BARKER 2005[[9](#_ENREF_9)] | FM | UK | NK | 30 | SEMI STRUCTURED INTERVIEWS AND FOCUS GROUP | NK |
| BATH & JANZEN 2012[[10](#_ENREF_10)] | LBP & SCIATICA | CANADA | 20-79 | 115 | QUESTIONNAIRE | THEMATIC ANALYSIS |
| BEATON ET AL. 2001[[11](#_ENREF_11)] | MSK (UPPERLIMB) | CANADA | NK | 24 | INTERVIEW | GROUNDED THEORY |
| BENJAMINSSON ET AL. 2007[[12](#_ENREF_12)] | CBP | SWEDEN | 15-64 | 17 | SEMI-STRUCTURED INTERVIEW | PHENOMENOLOGY |
| BORKAN ET AL. 1995[[13](#_ENREF_13)] | CBP | ISRAEL | 18-67 | 76 | INTERVIEWS, FOCUS GROUPS, OBSERVATION | CONTENT ANALYSIS |
| BOWMAN 1991[[14](#_ENREF_14)] | CBP | USA | 27-70 | 15 | IN-DEPTH INTERVIEW | PHENOMENOLOGY |
| BOWMAN 1994[[15](#_ENREF_15)] | See Bowman 1991 |  |  |  |  |  |
| BOWMAN 1994[[16](#_ENREF_16)] | See Bowman 1991 |  |  |  |  |  |
| BUSCH 2005[[17](#_ENREF_17)] | CBP | SWEDEN | 26-59 | 30 | SEMI-STRUCTURED INTERVIEW | GROUNDED THEORY |
| CAMPBELL 2007[[18](#_ENREF_18)] | CBP | UK | 34-78 | 16 | FOCUS GROUPS | THEMATIC ANALYSIS |
| CAMPBELL 2008[[19](#_ENREF_19)] | MSK | UK | 36-66 | 12 | INTERVIEWS | THEMATIC ANALYSIS |
| CARNES & UNDERWOOD 2008[[20](#_ENREF_20)] | CBP | UK | 24-83 | 13 | INTERVIEWS, FOCUS GROUPS, OBSERVATION | FRAMEWORK APPROACH |
| CHEW & MAY 1997[[21](#_ENREF_21)] | CBP | UK | 21-56 | 20 | INTERVIEWS, FOCUS GROUPS, OBSERVATION | GROUNDED THEORY |
| CLEMENCE & SEAMARK 2003[[22](#_ENREF_22)] | MSK | UK | NK | 6 | SEMI STRUCTURED INTERVIEW | GROUNDED THEORY |
| COOK 2000[[23](#_ENREF_23)] | CBP | UK | 22-63 | 7 | SEMI-STRUCTURED INTERVIEW | THEMATIC ANALYSIS |
| COOLE 2010A[[24](#_ENREF_24)] | CBP | UK | 22-58 | 25 | SEMI-STRUCTURED INTERVIEW | THEMATIC ANALYSIS |
| COOLE 2010B[[25](#_ENREF_25)] | See Coole 2010A |  |  |  |  |  |
| COOLE 2010C[[26](#_ENREF_26)] | See Coole 2010A |  |  |  |  |  |
| COOPER 2008[[27](#_ENREF_27)] | CBP | UK | 18-65 | 25 | SEMI-STRUCTURED INTERVIEW | FRAMEWORK ANALYSIS |
| COOPER 2009* [[28](#_ENREF_28)] | CBP | UK | 18-65 |  | SEMI-STRUCTURED INTERVIEW | FRAMEWORK ANALYSIS |
| CORBETT ET AL. 2007[[29](#_ENREF_29)] | CBP | UK | 19-59 | 6 | SEMI-STRUCTURED INTERVIEW | THEMATIC ANALYSIS |
| COX ET AL. 2003A[[30](#_ENREF_30)] | ENDOMETRIOSIS | AUSTRALIA | 20-64 | 61 | FOCUS GROUPS | THEMATIC ANALYSIS |
| COX ET AL. 2003B[[31](#_ENREF_31)] | See Cox et al 2003A |  |  |  |  |  |
| CROWE 2010A [[32](#_ENREF_32)] | CBP | NZ | 25-80 | 64 | SEMI-STRUCTURED INTERVIEW | THEMATIC ANALYSIS |
| CROWE 2010B [[33](#_ENREF_33)] | See Cox et al 2010A |  |  |  |  |  |
| CUDNEY, ET AL. 2002[[34](#_ENREF_34)] | FM | USA | 38-55 | 10 | UNSTRUCTURED CONVERSATIONS | CONTENT ANALYSIS |
| CUNNINGHAM 2006 [[35](#_ENREF_35)] | FM | CANADA | 30-70 | 8 | SEMI-STRUCTURED INTERVIEW | THEMATIC ANALYSIS |
| DE SOUZA & FRANK 2000[[36](#_ENREF_36)] | CBP | UK | 27-79 | 11 | INTERVIEWS, FOCUS GROUPS, OBSERVATION | FRAMEWORK APPROACH |
| DE SOUZA & FRANK 2007[[37](#_ENREF_37)] | CBP | UK | 27-79 | 11 | UNSTRUCTURED INTERVIEWS | THEMATIC ANALYSIS |
| DE SOUZA 2011 [[38](#_ENREF_38)] | See De Souza 2007 |  |  |  |  |  |
| DE VRIES 2011 [[39](#_ENREF_39)] | FM & MSK | NETHERLANDS | 31-60 | 21 | SEMI-STRUCTURED INTERVIEW | THEMATIC ANALYSIS |
| DEAN ET AL. 2005[[40](#_ENREF_40)] | LBP & SCIATICA | UK | 28-59 | 9 | SEMI-STRUCTURED INTERVIEW | IPA |
| DEAN ET AL. 2011[[41](#_ENREF_41)] | LBP | NEW ZEALAND | 27-61 | 33 | QUESTIONNAIRE & SEMI-STRUCTURED INTERVIEWS | MIXED METHODS |
| DENNY 2004A [[42](#_ENREF_42)] | ENDOMETRIOSIS | UK | 20-47 | 15 | IN-DEPTH INTERVIEWS | THEMATIC ANALYSIS |
| DENNY 2004B[[43](#_ENREF_43)] | ENDOMETRIOSIS | UK | 20-47 | 20 | IN-DEPTH INTERVIEWS, | THEMATIC ANALYSIS |
| DENNY 2009[[44](#_ENREF_44)] | ENDOMETRIOSIS | UK | 19-44 | 30 | SEMI-STRUCTURED INTERVIEWS, | NARRATIVE ANALYSIS |
| DENNY AND MANN 2007[[45](#_ENREF_45)] | See Denny 2009 |  |  |  |  |  |
| DENNY AND MANN 2008[[46](#_ENREF_46)] | See Denny 2009 |  |  |  |  |  |
| DEWAR ET AL. 2003[[47](#_ENREF_47)] | NON MALIGNANT | CANADA | 15-80 | 53 | NOMINAL GROUP TECHNIQUE | CLASSIFICATION |
| DEWAR ET AL. 2009[[48](#_ENREF_48)] | NON MALIGNANT | CANADA, USA | 40-65 | 19 | INTERVIEWS | CONSTANT COMPARATIVE METHODS |
| DICKSON 2003 [[49](#_ENREF_49)] | MSK | USA | 63-80 | 7 | INTERVIEWS & OBSERVATION | THEMATIC ANALYSIS |
| DRAGESUND 2008 [[50](#_ENREF_50)] | MSK | NORWAY | 26-68 | 13 | FOCUS GROUPS | THEMATIC ANALYSIS |
| EMAD 2006[[51](#_ENREF_51)] | ENDOMETRIOSIS | USA | 20S AND 30S | 17 | ON-LINE NARRATIVES, | NARRATIVE ANALYSIS |
| ESPELAND ET AL. 2001[[52](#_ENREF_52)] | LBP & SCIATICA | NORWAY | 14-91 | 99 | QUESTIONNAIRE AND SEMI-STRUCTURED INTERVIEWS | TEMPLATE ANALYSIS |
| EVANS ET AL. 2003[[53](#_ENREF_53)] | LBP & SCIATICA | USA | 49 (MEAN 9.2) | 31 | SEMI-STRUCTURED INTERVIEW | CONTENT ANALYSIS |
| GANNIK & JESPERSEN 1984[[54](#_ENREF_54)] | CBP | DENMARK | 20-54 | 60 | SEMI STRUCTURED INTERVIEW | BIOGRAPHICAL AND THEMATIC ANALYSIS |
| GILMOUR ET AL. 2008[[55](#_ENREF_55)] | ENDOMETRIOSIS | NZ | 16-45 | 18 | UNSTRUCTURED INTERVIEWS | FEMINIST RESEARCH |
| GLENTON 2003[[56](#_ENREF_56)] | CBP | NORWAY | 25-65 | 19 | INTERVIEWS | THEMATIC ANALYSIS |
| GRACE & MACBRIDE-STEWART 2007A[[57](#_ENREF_57)] | PELVIC | NEW ZEALAND | 22-51 | 42 | OPEN ENDED INTERVIEWS | PHENOMENOLOGY |
| GRACE & MACBRIDE-STEWART 2007B[[58](#_ENREF_58)] | PELVIC | NEW ZEALAND | 22-51 | 40 | OPEN ENDED INTERVIEWS | PHENOMENOLOGY |
| GRACE 1995[[59](#_ENREF_59)] | PELVIC | NEW ZEALAND | NK | 36 | FOCUS GROUPS | METADISCOURSE |
| GULLACKSEN 2004 [[60](#_ENREF_60)] | FM & MSK | SWEDEN | 23-55 | 18 | IN-DEPTH INTERVIEWS | PHENOMENOLOGY |
| GUSTAFSSON 2004 [[61](#_ENREF_61)] | FM & MSK | SWEDEN | 23-59 | 18 | SEMI-STRUCTURED INTERVIEW | GROUNDED THEORY |
| HALLBERG 1998 [[62](#_ENREF_62)] | FM | SWEDEN | 22-60 | 22 | SEMI-STRUCTURED INTERVIEW | GROUNDED THEORY |
| HALLBERG 2000* [[63](#_ENREF_63)] | See Hallberg 1998 |  |  |  |  |  |
| HARDING 2005 [[64](#_ENREF_64)] | MSK | UK | 29-71 | 15 | IN-DEPTH INTERVIEWS | FRAMEWORK ANALYSIS |
| HATCHETT ET AL. 2009[[65](#_ENREF_65)] | PELVIC (MALE) | USA | 22-76 | 47 | OPEN ENDED QUESTIONS IN A JOURNAL | GROUNDED THEORY |
| HELLSTROM 1999 [[66](#_ENREF_66)] | FM | SWEDEN | 32-50 | 10 | IN-DEPTH INTERVIEWS | PHENOMENOLOGY |
| HENRIKSSON 1995A [[67](#_ENREF_67)] | FM | USA AND SWEDEN | 16-57 | 40 | SEMI STRUCTURED INTERVIEWS AND FOCUS GROUP | CONTENT ANALYSIS |
| HENRIKSSON, 1995B[[68](#_ENREF_68)] | See Henriksson 1995A |  |  |  |  |  |
| HOLLOWAY 2007 [[69](#_ENREF_69)] | CBP | UK | 28-62 | 18 | SEMI-STRUCTURED INTERVIEW | IPA |
| HOLLOWAY ET AL. 2000[[70](#_ENREF_70)] | CBP | UK | 28-80 | 20 | UNSTRUCTURED INTERVIEWS | IPA |
| HOOPER & ONG 2005[[71](#_ENREF_71)] | LBP | UK | NK | 5 | INTERVIEW | THEMATIC AND NARRATIVE METHODS |
| HUNHAMMAR 2009 [[72](#_ENREF_72)] | MSK | SWEDEN | 19-58 | 15 | IN-DEPTH INTERVIEWS | GROUNDED THEORY |
| HUNTINGTON AND GILMOUR 2005[[73](#_ENREF_73)] | ENDOMETRIOSIS | NZ | 16-46 | 19 | UNSTRUCTURED INTERVIEWS | FEMINIST RESEARCH |
| HUSH ET AL. 2009[[74](#_ENREF_74)] | CBP | AUSTRALIA | 31-59 | 36 | FOCUS GROUPS | FRAMEWORK APPROACH |
| HUSH ET. AL 2010[[75](#_ENREF_75)] | See Hush et al. 2009 |  |  |  |  |  |
| JOHANSSON 1996[[76](#_ENREF_76)] | MSK | SWEDEN | 21-60 | 20 | SEMI-STRUCTURED INTERVIEW | GROUNDED THEORY |
| JOHANSSON 1997[[77](#_ENREF_77)] | See Johansson 1996 |  |  |  |  |  |
| JOHANSSON 1999 [[78](#_ENREF_78)] | See Johansson 1996 |  |  |  |  |  |
| JOHSSON AND HEDELIN 2008[[79](#_ENREF_79)] | PELVIC (MALE) | SWEDEN | 30-62 | 10 | INTERVIEWS | PHENOMENOLOGY |
| JONES ET AL. 2004[[80](#_ENREF_80)] | ENDOMETRIOSIS | UK | 21-44 | 24 | SEMI-STRUCTURED INTERVIEWS, | GROUNDED THEORY |
| KEEN ET AL. 1999[[81](#_ENREF_81)] | LBP | UK | 18-60 | 27 | INTERVIEW | FRAMEWORK ANALYSIS |
| KELLEY 1997 [[82](#_ENREF_82)] | FM | USA | 50 (MEAN) | 22 | IN-DEPTH INTERVIEWS | NARRATIVE ANALYSIS |
| KUGELMANN 1999 [[83](#_ENREF_83)] | NON MALIGNANT | USA | 20-50 | 14 | INTERVIEWS | PHENOMENOLOGY |
| LACHAPELLE 2008 [[84](#_ENREF_84)] | FM & MSK | CANADA | 23-75 | 45 | ETHNOGRAPHY AND FOCUS GROUPS | ETHNOGRAPHY |
| LAERUM ET AL 2006 [[85](#_ENREF_85)] | CBP | UK | 23-65 | 35 | SEMI-STRUCTURED INTERVIEWS | TEMPLATE ANALYSIS |
| LAMBERT ET AL. 2000[[86](#_ENREF_86)] | ARTHRITIS | USA | NK | 14 | FOCUS GROUPS | NK |
| LAYZELL 2001[[87](#_ENREF_87)] | LBP | UK | NK | NK | QUESTIONNAIRE / FOCUS GROUP | COMBINED METHODOLOGICAL APPROACH |
| LEMPP 2009 [[88](#_ENREF_88)] | FM | UK | 20-69 | 12 | SEMI-STRUCTURED INTERVIEW | THEMATIC ANALYSIS |
| LIDDLE 2007 [[89](#_ENREF_89)] | CBP | N IRELAND | 20-65 | 18 | FOCUS GROUPS | THEMATIC ANALYSIS |
| LIEDBERG 2002 [[90](#_ENREF_90)] | FM | SWEDEN | 26-64 | 39 | IN-DEPTH INTERVIEWS | THEMATIC ANALYSIS |
| LILLRANK 2003[[91](#_ENREF_91)] | CBP | FINLAND | 20-66 | 30 | ESSAYS | RICOEUR INTERPRETIVE THEORY |
| LOFGREN 2006 [[92](#_ENREF_92)] | FM | SWEDEN | 30-63 | 12 | DIARIES, FGS, INTERVIEWS | GROUNDED THEORY |
| LUNDBERG 2007 [[93](#_ENREF_93)] | MSK | SWEDEN | 30-64 | 10 | IN-DEPTH INTERVIEWS | PHENOMENOLOGY |
| MADDEN 2006 [[94](#_ENREF_94)] | FM | UK | 25-55 | 17 | SEMI-STRUCTURED INTERVIEW | INDUCTION/ ABDUCTION |
| MAGNUSSEN ET AL. 2007[[95](#_ENREF_95)] | CBP | NORWAY | 38-56 | 17 | FOCUS GROUPS | HERMENEUTIC APPROACH |
| MANDERSON ET AL. 2008[[96](#_ENREF_96)] | ENDOMETRIOSIS | AUSTRALIA | 20-78 | 40 | IN-DEPTH INTERVIEWS | GROUNDED THEORY |
| MANNERKORPI 1999 [[97](#_ENREF_97)] | FM | SWEDEN | 29-59 | 11 | IN-DEPTH INTERVIEWS | PHENOMENOLOGY |
| MARKOVIC ET AL. 2008[[98](#_ENREF_98)] | ENDOMETRIOSIS | AUSTRALIA | 20-78 | 30 | IN-DEPTH INTERVIEWS | GROUNDED THEORY |
| MASON 2004[[99](#_ENREF_99)] | CBP | UK | NK | 5 | SEMI-STRUCTURED INTERVIEWS | IPA |
| MAY ET AL.2000[[100](#_ENREF_100)] | CBP | UK | 20-55 | 12 | SEMI-STRUCTURED INTERVIEW | CONTENT ANALYSIS |
| MAY 2001[[101](#_ENREF_101)] | CBP | UK | 29-77 | 34 | INTERVIEWS, FOCUS GROUPS, OBSERVATION | FRAMEWORK APPROACH |
| MAY 2007[[102](#_ENREF_102)] | See May 2001 |  |  |  |  |  |
| MCGOWAN ET AL. 2007[[103](#_ENREF_103)] | PELVIC | UK | 21-50 | 32 | WRITTEN STORIES, | PHENOMENOLOGY |
| MCINTOSH & SHAW 2003[[104](#_ENREF_104)] | LBP & SCIATICA | UK | 25-64 | 37 | FOCUS GROUP | FRAMEWORK ANALYSIS |
| MCPHILLIPS -TANGUM ET AL. 1998 [[105](#_ENREF_105)] | CBP | USA | 25-65 | 54 | INTERVIEWS, FOCUS GROUPS, OBSERVATION | CONTENT ANALYSIS |
| MENGSHOEL [[106](#_ENREF_106)] 2004 [[106](#_ENREF_106)] | FM | NORWAY | 37-49 | 5 | SEMI-STRUCTURED INTERVIEW | THEMATIC ANALYSIS |
| MOORE ET AL. 2002[[107](#_ENREF_107)] | PELVIC | UK | NK | 20 | SEMI-STRUCTURED INTERVIEWS | CONSTANT COMPARISON |
| MORRIS 2004[[108](#_ENREF_108)] | LBP | UK | 38-61 | 6 | INTERVIEWS, OBSERVATION | NK |
| ONG & HOOPER 2003[[109](#_ENREF_109)] | LBP | UK | NK | 6 | FOCUS GROUP | THEMATIC ANALYSIS |
| ONG & HOOPER 2006 [[110](#_ENREF_110)] | CBP | UK | 30-59 | 2 | OPEN INTERVIEWS | MATCHED PAIR DESIGN |
| ONG ET AL. 2004[[111](#_ENREF_111)] | LBP | UK | 30-59 | 16 | INTERVIEW | THEMATIC ANALYSIS |
| ONG ET AL. 2011[[112](#_ENREF_112)] | LBP & SCIATICA | UK | 19-59 | 37 | SEMI-STRUCTURED INTERVIEWS | CONSTANT COMPARISON |
| OSBORN 1998 [[113](#_ENREF_113)] | CBP | UK | 25-55 | 9 | SEMI-STRUCTURED INTERVIEW | IPA |
| OSBORN 2006[[114](#_ENREF_114)] | CBP | UK | 36-52 | 6 | SEMI-STRUCTURED INTERVIEW | IPA |
| OSBORN 2008 [[115](#_ENREF_115)] | CBP | UK | 36-52 | 10 | SEMI-STRUCTURED INTERVIEW | IPA |
| PATEL 2007 [[116](#_ENREF_116)] | MSK | UK | 29-62 | 38 | SEMI-STRUCTURED INTERVIEW | THEMATIC ANALYSIS |
| PAULSON 2001[[117](#_ENREF_117)] | FM | SWEDEN | 41-56 | 14 | NARRATIVE INTERVIEW | PHENOMENOLOGY |
| PAULSON 2002A [[118](#_ENREF_118)] | See Paulson 2001 |  |  |  |  |  |
| PAULSON 2002B [[119](#_ENREF_119)] | See Paulson 2001 |  |  |  |  |  |
| PRICE ET AL. 2006[[120](#_ENREF_120)] | PELVIC | UK | 20-59 | 26 | SEMI-STRUCTURED INTERVIEW, | GROUNDED THEORY |
| RAAK & WAHREN 2006[[121](#_ENREF_121)] | CBP | SWEDEN | WORKING AGE | 10 | IN-DEPTH INTERVIEW | CONTENT ANALYSIS |
| RAHEIM 2006 [[122](#_ENREF_122)] | FM | NORWAY | 34-51 | 12 | LIFE FORM INTERVIEWS | PHENOMENOLOGY |
| RAYMOND 2000 [[123](#_ENREF_123)] | FM | CANADA | 38-47 | 7 | SEMI-STRUCTURED INTERVIEW | THEMATIC ANALYSIS |
| REID 2004[[124](#_ENREF_124)] | CBP | UK | 17-65 | 50 | INTERVIEWS | THEMATIC ANALYSIS |
| RHODES 1999 [[125](#_ENREF_125)] | CBP | USA | 25-65 | 54 | IN-DEPTH INTERVIEWS | THEMATIC ANALYSIS |
| ROGERS 1999[[126](#_ENREF_126)] | LBP | AUSTRALIA | 28-70 | 17 | SEMI STRUCTURED INTERVIEW | NK |
| ROSS ET AL.2001 [[127](#_ENREF_127)] | MSK | CANADA | 60+ | 50 | FOCUS GROUPS AND MAIL-BACK QUESTIONNAIRES | NK |
| SALLINEN 2010 [[128](#_ENREF_128)] | FM | FINLAND | 34-65 | 20 | NARRATIVE INTERVIEW | THEMATIC ANALYSIS |
| SALLINEN 2011 [[129](#_ENREF_129)] | See Sallinen 2011 |  |  |  |  |  |
| SANDERS 2002 [[130](#_ENREF_130)] | MSK | UK | 51-91 | 27 | IN-DEPTH INTERVIEWS | GROUNDED THEORY |
| SATINK ET AL.2004[[131](#_ENREF_131)] | CBP | NETHERLANDS | 42-70 | 7 | SEMI-STRUCTURED INTERVIEW | NARRATIVE APPROACH |
| SAVIDGE ET AL. 1998[[132](#_ENREF_132)] | PELVIC | UK | 21-61 | 21 | SEMI-STRUCTURED INTERVIEWS | NK |
| SCHAEFER 1995[[133](#_ENREF_133)] | FM | USA | NK | 36 | INTERVIEW | CONSTANT COMPARATIVE METHOD |
| SCHAEFER 1997[[134](#_ENREF_134)] | FM | USA | 27–46 | 8 | NARRATIVE DIARY | VAN MANEN’S METHODS |
| SCHAEFER 2005 [[135](#_ENREF_135)] | FM | USA | 37-59 | 10 | IN-DEPTH INTERVIEWS | PHENOMENOLOGY |
| SCHERS ET AL. 2001[[136](#_ENREF_136)] | LBP & SCIATICA | NETHERLANDS | 25-68 | 20 | SEMI-STRUCTURED INTERVIEW | CATEGORISED |
| SEEAR 2009A[[137](#_ENREF_137)] | ENDOMETRIOSIS | AUSTRALIA | 24-55 | 20 | SEMI STRUCTURED INTERVIEWS | THEMATIC ANALYSIS |
| SEEAR 2009B[[138](#_ENREF_138)] | See Seear 2009A |  |  |  |  |  |
| SEEAR 2009C[[139](#_ENREF_139)] | See Seear 2009A |  |  |  |  |  |
| SEERS & FRIEDLI 1996[[140](#_ENREF_140)] | NON MALIGNANT | UK | 21-86 | 75 | INTERVIEWS | THEMATIC CODING |
| SKELTON 1998[[141](#_ENREF_141)] | LBP | UK | 18-66 | 52 | SEMI STRUCTURED INTERVIEW | THEMATIC ANALYSIS |
| SKELTON ET AL. 1995[[142](#_ENREF_142)] | See Skelton 1998 |  |  |  |  |  |
| SKELTON ET AL. 1996[[143](#_ENREF_143)] | See Skelton 1998 |  |  |  |  |  |
| SKULADOTTIR 2011 [[144](#_ENREF_144)] | MSK | ICELAND | 35-55 | 5 | IN-DEPTH INTERVIEWS | GROUNDED THEORY |
| SLADE 2009A[[145](#_ENREF_145)] | CBP | AUSTRALIA | 26–64 | 18 | FOCUS GROUPS | GROUNDED THEORY |
| SLADE 2009B[[146](#_ENREF_146)] | See Slade 2009A |  |  |  |  |  |
| SLADE ET AL. 2009C[[147](#_ENREF_147)] | See Slade 2009A |  |  |  |  |  |
| SLOOTS 2010[[148](#_ENREF_148)] | LBP | NETHERLANDS | 29-57 | 23 | SEMI-STRUCTURED INTERVIEWS | CONSTANT COMPARISON |
| SMITH 2007[[149](#_ENREF_149)] | CBP | UK | 36-52 | 6 | SEMI-STRUCTURED INTERVIEW | IPA |
| SNELGROVE 2009 [[150](#_ENREF_150)] | CBP | UK | 39-66 | 10 | SEMI-STRUCTURED INTERVIEW | IPA |
| SNELGROVE ET AL 2013[[151](#_ENREF_151)] | CBP | UK | 40-76 | 10 | SEMI-STRUCTURED INTERVIEWS | IPA |
| SODERBERG & NORBERG 1995[[152](#_ENREF_152)] | FM | SWEDEN | 35-50 | 14 | INTERVIEW | PHENOMENOLOGY |
| SODERBERG 1999 [[153](#_ENREF_153)] | See Soderberg & Norberg 1995 |  |  |  |  |  |
| SODERBERG 2001 [[154](#_ENREF_154)] | FM | SWEDEN | 35-60 | 25 | IN-DEPTH INTERVIEWS | PHENOMENOLOGY |
| SODERBERG 2002[[155](#_ENREF_155)] | See Soderberg 2001 |  |  |  |  |  |
| SOFAERBENNETT ET AL. 2007[[156](#_ENREF_156)] | CBP | UK | 60-84 | 16 | SEMI-STRUCTURED INTERVIEWS | IPA |
| SOKUNBI ET AL. 2010[[157](#_ENREF_157)] | CBP | UK | 29-56 | 9 | FOCUS GROUPS | CONTENT ANALYSIS |
| STEEN 2001 [[158](#_ENREF_158)] | MSK | NORWAY | ADULTS | 48 | SEMI-STRUCTURED INTERVIEW | PHENOMENOLOGY |
| STRONG 1994 [[159](#_ENREF_159)] | CBP | AUSTRALIA | 30-75 | 7 | FOCUS GROUPS | THEMATIC ANALYSIS |
| STRONG 1995 [[160](#_ENREF_160)] | See Strong 1994 |  |  |  |  |  |
| STRUNIN & BODEN 2004[[161](#_ENREF_161)] | CBP | USA | WORK AGE | 414 | SEMI-STRUTURED PHONE INTERVIEWS | NOT STATED |
| STRZEMPKO AND CHESLA 2007[[162](#_ENREF_162)] | ENDOMETRIOSIS | USA | 23-50 | NK | INDIVIDUAL AND PARTNER INTERVIEWS | INTERPRETIVE PHENOMENOLOGY |
| STURGEJACOBS 2002 [[163](#_ENREF_163)] | FM | CANADA | 20-57 | 9 | UNSTRUCTURED INTERVIEW | PHENOMENOLOGY |
| TARASUK & EAKIN 1995[[164](#_ENREF_164)] | LBP | CANADA | 21-62 | 15 | SEMI-STRUCTURED INTERVIEWS | GROUNDED THEORY |
| TAVAFIAN ET AL. 2008[[165](#_ENREF_165)] | CBP | IRAN | 18-70 | 24 | FOCUS GROUPS | THEMATIC ANALYSIS |
| TEH 2009 [[166](#_ENREF_166)] | CBP | USA | 63-86 | 15 | IN-DEPTH INTERVIEWS | GROUNDED THEORY |
| THOMAS 2000[[167](#_ENREF_167)] | NON MALIGNANT | USA | 27-79 | 13 | NONDIRECTIVE PHENOMENOLOGICAL INTERVIEWS | EIDETIC PHENOMENOLOGY |
| THORNE ET AL. 2004[[168](#_ENREF_168)] | FM | CANADA | 33-54 | 12 | INTERVIEWS AND FOCUS GROUP | INDUCTIVE THEMATIC ANALYSIS |
| TOYE 2010[[169](#_ENREF_169)] | CBP | UK | 29-67 | 20 | SEMI-STRUCTURED INTERVIEW | GROUNDED THEORY |
| TOYE 2012A[[170](#_ENREF_170)] | See Toye 2010 |  |  |  |  |  |
| TOYE 2012B [[171](#_ENREF_171)] | See Toye 2010 |  |  |  |  |  |
| TVEITO ET AL. 2010[[172](#_ENREF_172)] | LBP | USA | 23-66 | 38 | FOCUS GROUPS | CONSTANT COMPARISON GUIDED BY GROUNDED THEORY |
| UNDELAND 2007 [[173](#_ENREF_173)] | FM | NORWAY | 42-67 | 11 | FOCUS GROUPS | THEMATIC ANALYSIS |
| UNDERWOOD ET AL. 2006[[174](#_ENREF_174)] | LBP & SCIATICA | UK | 18-64 | 1259 | FREE-TEXT QUESTIONNAIRE | FRAMEWORK APPROACH |
| VROMAN 2009[[175](#_ENREF_175)] | CBP | USA | 19-83 | 133 | OPEN ENDED QUESTIONNAIRE DATA | THEMATIC ANALYSIS |
| WADE & SHANTALL 2003[[176](#_ENREF_176)] | CBP | SOUTH AFRICA | NK | 3 | UNSTRUCTURED INTERVIEWS | PHENOMENOLOGY |
| WALKER 1999[[177](#_ENREF_177)] | CBP | UK | 28-80 | 20 | IN-DEPTH INTERVIEWS | PHENOMENOLOGY |
| WALKER 2006 [[178](#_ENREF_178)] | See Walker 1999 |  |  |  |  |  |
| WARWICK ET AL. 2004 [[179](#_ENREF_179)] | PELVIC | UK | 21-61 | 8 | SEMI-STRUCTURED INTERVIEWS, | INTERPRETIVE PHENOMENOLOGICAL ANALYSIS |
| WERNER 2003A [[180](#_ENREF_180)] | MSK | NORWAY | 26–58 | 10 | IN-DEPTH INTERVIEWS | PHENOMENOLOGY |
| WERNER 2003B [[181](#_ENREF_181)] | MSK | NORWAY | 31-53 | 6 | IN-DEPTH INTERVIEWS | PHENOMENOLOGY |
| WERNER 2004 [[182](#_ENREF_182)] | See Werner 2003B |  |  |  |  |  |
| WHELAN 2007[[183](#_ENREF_183)] | ENDOMETRIOSIS | CANADA | NK | NK | FOCUS GROUPS AND MAILING LIST STORIES | GROUNDED THEORY |
| WHITE & SEIBOLD 2008[[184](#_ENREF_184)] | CBP | AUSTRALIA | 32-44 | 5 | OPEN-ENDED INTERVIEWS | NARRATIVE AUTOETHNOGRAPHY |
| WHITNEY 1998[[185](#_ENREF_185)] | ENDOMETRIOSIS | USA | NK | 46 | WRITTEN RESPONSES ON QUESTIONNAIRE | NK |
| YOUNG ET AL.2011[[186](#_ENREF_186)] | CBP | CANADA | 20-65 | 31 | FOCUS GROUPS | PHENOMENOLOGY |
| ZADINSKY & BOYLE 1996[[187](#_ENREF_187)] | PELVIC | USA | 19-52 | 14 | SEMI-STRUCTURED INTERVIEWS & DIARIES, | CONSTANT COMPARISON |

**References to Appendix 1**

1. Aegler B, Satink T: **Performing occupations under pain: the experience of persons with chronic pain**. *Scandinavian Journal of Occupational Therapy* 2009, **16**(1):49-56.

2. Afrell M, Biguet G, Rudebeck CE: **Living with a body in pain -- between acceptance and denial**. *Scandinavian Journal of Caring Sciences* 2007, **21**(3):291-296.

3. Allegretti A, Borkan J, Reis S, Griffiths F: **Paired interviews of shared experiences around chronic low back pain: classic mismatch between patients and their doctors**. *Family Practice* 2010, **27**(6):676-683.

4. Arnold LM, Crofford LJ, Mease PJ, Burgess SM, Palmer SC, Abetz L, Martin SA: **Patient perspectives on the impact of fibromyalgia**. *Patient Education & Counseling* 2008, **73**(1):114-120.

5. Ashby S, Richards K, James C: **The effect of fear of movement on the lives of people with chronic low back pain**. *International Journal of Therapy and Rehabilitation* 2010, **17**(5):232-239.

6. Bair MJ, Matthias MS, Nyland KA, Huffman MA, Stubbs DL, Kroenke K, Damush TM: **Barriers and facilitators to chronic pain self-management: a qualitative study of primary care patients with comorbid musculoskeletal pain and depression**. *Pain Medicine* 2009, **10**(7):1280-1290.

7. Ballard K, Lowton K, Wright J: **What’s the delay? A qualitative study of women’s experiences of reaching a diagnosis of endometriosis**. *Fertility and Sterility* 2006, **86**(5):1296-1301.

8. Ballweg ML: **Endometriosis: the patient's persective**. *Infertility and Reproductive Medicine Clinics of North America* 1992, **3**:747-761.

9. Barker KK: **The fibromyalgia story: Medical authority and women’s world of pain**. Philadelphia, USA: Temple University Press; 2005.

10. Bath B, Janzen B: **Patient and referring health care provider satisfaction with a physiotherapy spinal triage assessment service**. *Journal of multidisciplinary healthcare* 2012, **5**:1-15.

11. Beaton DE, Tarasuk V, Katz JN, Wright JG, Bombardier C: **"Are you better?" A qualitative study of the meaning of recovery**. *Arthritis & Rheumatism: Arthritis Care & Research* 2001, **45**(3):270-280.

12. Benjaminsson O, Biguet G, Arvidsson I, Nilsson-Wikmar L: **Recurrent low back pain: relapse from a patients perspective**. *Journal of Rehabilitation Medicine* 2007, **39**(8):640-645.

13. Borkan J, Reis S, Hermoni D, Biderman A: **Talking about the pain: a patient-centered study of low back pain in primary care**. *Social Science and Medicine* 1995, **40**(7):977-988.

14. Bowman JM: **The meaning of chronic low back pain**. *AAOHN Journal* 1991, **39**(8):381-384.

15. Bowman JM: **Reactions to chronic low back pain**. *Issues in Mental Health Nursing* 1994, **15**(4):445-453.

16. Bowman JM: **Experiencing the chronic pain phenomenon: a study**. *Rehabilitation Nursing* 1994, **19**(2):91-95.

17. Busch H: **Appraisal and coping processes among chronic low back pain patients**. *Scandinavian Journal of Caring Sciences* 2005, **19**(4):396-402.

18. Campbell C, Guy A: **'Why can't they do anything for a simple back problem?' A qualitative examination of expectations for low back pain treatment and outcome**. *Journal of Health Psychology* 2007, **12**(4):641-652.

19. Campbell C, Cramb G: **'Nobody likes a back bore'--exploring lay perspectives of chronic pain: revealing the hidden voices of nonservice users**. *Scandinavian Journal of Caring Sciences* 2008, **22**(3):383-390.

20. Carnes D, Anwer Y, Underwood M, Harding G, Parson S: **Influences on older people's decision amking regarding choice of topical or oral NSAIDs for knee pain: qualitative study**. *BMJ* 2008, **336**:142-145.

21. Chew CA, May CR: **The benefits of back pain**. *Family Practice* 1997, **14**(6):461-465.

22. Clemence ML, Seamark DA: **GP referral for physiotherapy to musculoskeletal conditions—a qualitative study**. *Family Practice* 2003, **20**:578-582.

23. Cook FM, Hassenkamp AM: **Active rehabilitation for chronic low back pain: The patients' perspective**. *Physiotherapy (London)* 2000, **86**(2):61-68.

24. Coole C, Watson PJ, Drummond A: **Staying at work with back pain: patients' experiences of work-related help received from GPs and other clinicians. A qualitative study**. *BMC Musculoskeletal Disorders* 2010a, **11**.

25. Coole C, Drummond A, Watson PJ, Radford K: **What concerns workers with low back pain? Findings of a qualitative study of patients referred for rehabilitation**. *Journal of Occupational Rehabilitation* 2010b, **20**(4):472-480.

26. Coole C, Watson PJ, Drummond A: **Low back pain patients' experiences of work modifications; a qualitative study**. *BMC Musculoskeletal Disorders* 2010c, **11**.

27. Cooper K, Smith BH, Hancock E: **Patient-centredness in physiotherapy from the perspective of the chronic low back pain patient**. *Physiotherapy (London)* 2008, **94**(3):244-252.

28. Cooper K, Smith BH, Hancock E: **Patients' perceptions of self-management of chronic low back pain: evidence for enhancing patient education and support**. *Physiotherapy* 2009, **95**(1):43-50.

29. Corbett M, Foster NE, Ong BN: **Living with low back pain-- stories of hope and despair**. *Social Science & Medicine* 2007, **65**(8):1584-1595.

30. Cox H, Henderson L, Andersen N, Cagliarini G, Ski C: **Focus group study of endometriosis:Struggle, loss and the medical merry-go-round**. *International Journal of Nursing Practice* 2003a, **9**:2-9.

31. Cox H, Henderson L, Wood R, Cagliarini G: **Learning to take charge: women’s experiences of living with endometriosis**. *Complementary Therapies in Nursing & Midwifery* 2003b, **9**:62-68.

32. Crowe M, Whitehead L, Jo M, Baxter D, Panckhurst A: **Self-management and chronic low back pain: a qualitative study**. *Journal of Advanced Nursing* 2010a, **66**(7):1478-1486.

33. Crowe M, Whitehead L, Gagan MJ, Baxter GD, Pankhurst A, Valledor V: **Listening to the body and talking to myself - the impact of chronic lower back pain: a qualitative study**. *International Journal of Nursing Studies* 2010b, **47**(5):586-592.

34. Cudney SA, Butler MR, Weinert C, Sullivan T: **Ten rural women living with fibromyalgia tell it like it is**. *Holistic Nursing Practice* 2002, **16**(3):35-45.

35. Cunningham MM, Jillings C: **Individuals' descriptions of living with fibromyalgia**. *Clinical Nursing Research* 2006, **15**(4):258-273.

36. De Souza LHL, Frank AO: **Subjective pain experience of people with chronic back pain**. *Physiotherapy Research International* 2000, **5**(4):207-219.

37. De Souza LHL, Frank AO: **Experiences of living with chronic back pain: the physical disabilities**. *Disability & Rehabilitation* 2007, **29**(7):587-596.

38. De Souza L, Frank AO: **Patients' experiences of the impact of chronic back pain on family life and work**. *Disability & Rehabilitation* 2011, **33**(4):310-619.

39. de Vries H, Brouwer S, Groothoff JW, Geertzen JH, Reneman MF: **Staying at work with chronic nonspecific musculoskeletal pain: a qualitative study of workers' experiences**. *BMC Musculoskeletal Disorders* 2011, **12**.

40. Dean SG, Smith JA, Payne S, Weinman J: **Managing time: An interpretative phenomenological analysis of patients' and physiotherapists' perceptions of adherence to therapeutic exercise for low back pain**. *Disability and Rehabilitation* 2005, **27**(11):625-636.

41. Dean SG, Hudson S, Hay-Smith EJ, Milosavljevic S: **Rural workers' experience of low back pain: exploring why they continue to work**. *Journal of Occupational Rehabilitation* 2011, **21**(3):395-409.

42. Denny E: **Women’s experience of endometriosis**. *Journal of Advanced Nursing* 2004a, **46**(6):641-648.

43. Denny E: **‘You are one of the unlucky ones’: delay in the diagnosis of endometriosis**. *Diversity in Health and Social Care* 2004b, **1**:39-44.

44. Denny E: **I never know from one day to another how I will feel: pain and uncertainty in women with endometriosis**. *Qualitative Health Research* 2009, **19**(7):985-995.

45. Denny E, Mann CH: **Endometriosis-associated dyspareunia: the impact on women’s lives**. *Journal of Family Planning and Reproductive Health Care* 2007, **33**:189-193.

46. Denny E, Mann C: **Endometriosis and the primary care consultation**. *European Journal of Obstetrics & Gynecology and Reproductive Biology* 2008, **139**:111-115.

47. Dewar A, White M, Posade ST, Dillon W: **Using nominal group technique to assess chronic pain, patients' perceived challenges and needs in a community health region**. *Health Expectations* 2003, **6**(1):44-52.

48. Dewar AL, Gregg K, White MI, Lander J: **Navigating the health care system: perceptions of patients with chronic pain**. *Chronic Diseases in Canada* 2009, **29**(4):162-168.

49. Dickson GL, Kim JI: **Reconstructing a meaning of pain: older Korean American women's experiences with the pain of osteoarthritis**. *Qualitative Health Research* 2003, **13**(5):675-688.

50. Dragesund T, Råheim M: **Norwegian psychomotor physiotherapy and patients with chronic pain: Patients' perspective on body awareness**. *Physiotherapy Theory & Practice* 2008, **24**(4):243-255.

51. Emad MC: **At WITSENDO: Communal embodiment through storytelling in women's experiences with endometriosis**. *Women's Studies International Forum 29 (2006) 197–207* 2006, **29**:197-207.

52. Espeland A, Baerheim A, Albrektsen G, Korsbrekke K, Larsen JL: **Patients' views on importance and usefulness of plain radiography for low back pain**. *Spine* 2001, **26**(12):1356-1363.

53. Evans RL, Maiers MJ, Bronfort G: **What do patients think? Results of a mixed methods pilot study assessing sciatica patients’ interpretations of satisfaction and improvement**. *Journal of Manipulative and Physiological Therapeutics* 2003, **26**:502-509.

54. Gannik D, Jespersen M: **Lay concepts and strategies for handling symptoms of disease. A sample of adult men and women experiencing back pain symptoms**. *Scandinavian Journal of Primary Health Care* 1984, **2**(2):67-76.

55. Gilmour JA, Huntington A, Wilson HV: **The impact of endometriosis on work and social participation**. *International Journal of Nursing Practice* 2008 **14**:443-448.

56. Glenton C: **Chronic back pain sufferers--striving for the sick role**. *Social Science & Medicine* 2003, **57**(11):2243-2252.

57. Grace VM, MacBride-Stewart S: **'Women get this': gendered meanings of chronic pelvic pain**. *Health* 2007a, **11**(1):47-67.

58. Grace VM, MacBride-Stewart S: **"How to say it": women's descriptions of pelvic pain**. *Women & Health* 2007b, **46**(4):81-98.

59. Grace V: **Problems women patients experience in the medical encounter for chronic pelvic pain: A New Zealand study**. *Health Care for Women* 1995, **16**(6):509-519.

60. Gullacksen AC, Lidbeck J: **The life adjustment process in chronic pain: psychosocial assessment and clinical implications**. *Pain Research & Management* 2004, **9**(3):145-153.

61. Gustafsson M, Ekholm J, Ohman A: **From shame to respect: musculoskeletal pain patients' experience of a rehabilitation programme, a qualitative study**. *Journal of Rehabilitation Medicine* 2004, **36**(3):97-103.

62. Hallberg LR, Carlsson SG: **Psychosocial vulnerability and maintaining forces related to fibromyalgia: in-depth interviews with twenty-two female patients**. *Scandinavian Journal of Caring Sciences* 1998, **12**(2):95-104.

63. Hallberg LR, Carlsson SG: **Coping with fibromyalgia. A qualitative study**. *Scandinavian Journal of Caring Sciences* 2000, **14**(1):29-36.

64. Harding G, Parsons S, Rahman A, Underwood M: **"It struck me that they didn't understand pain": the specialist pain clinic experience of patients with chronic musculoskeletal pain**. *Arthritis & Rheumatism* 2005, **53**(5):691-696.

65. Hatchett L, FitzGerald MP, Potts J, Winder A, Mickelberg K, Barrell T, Kusek JW, Urological-Pelvic-Pain-Collaborative-Research-Network: **Life impact of Urological Pain Syndromes**. *Journal of Health Psychology* 2009, **14**(6):741-750.

66. Hellstrom O, Bullington J, Karlsson G, Lindqvist P, Mattsson B: **A phenomenological study of fibromyalgia. Patient perspectives**. *Scandinavian Journal of Primary Health Care* 1999, **17**(1):11-16.

67. Henriksson CM: **Living with continuous muscular pain - patient perspectives. Part I: encounters and consequences**. *Scandinavian Journal of Caring Sciences* 1995, **9**(2):67-76.

68. Henriksson CM: **Living with continuous muscular pain--patient perspectives. Part II: Strategies for daily life**. *Scandinavian Journal of Caring Sciences* 1995, **9**(2):77-86.

69. Holloway I, Sofaer-Bennett B, Walker J: **The stigmatisation of people with chronic back pain**. *Disability & Rehabilitation* 2007, **29**(18):1456-1464.

70. Holloway I, Sofaer B, Walker J: **The Transition from Well Person to "Pain Afflicted" Patient: The Career of People with Chronic Back Pain**. *Illness, Crisis, & Loss* 2000, **8**(4):373-387.

71. Hooper H, Ong BN: **When Harry Met Barry, and Other Stories: A Partner's Influence on Relationships in Back Pain Care**. *Anthropology & Medicine* 2005, **12**(1):47-60.

72. Hunhammar C, Nilsson-Wikmar L, Lofgren M: **Striving to master variable pain: An interview study in primary care patients with non-specific long-term neck/shoulder pain**. *Journal of Rehabilitation Medicine* 2009, **41**(9):768-774.

73. Huntington A, Gilmour JA: **A life shaped by pain: women and endometriosis**. *Journal of Clinical Nursing 14,* 2005, **14**:1124-1132.

74. Hush JM, Refshauge K, Sullivan G, De L, Maher CG, McAuley JH: **Recovery: what does this mean to patients with low back pain?** *Arthritis & Rheumatism* 2009, **61**(1):124-131.

75. Hush JM RK, Sullivan G, De Souza L, McAuley JH: : **Do numerical rating scales and the Roland-Morris Disability Questionnaire capture changes that are meaningful to patients with persistent back pain?** *Clinical Rehabilitation* 2010, **24**(7):648-657.

76. Johansson E, Hamberg K, Lindgren G, Westman G: **"I've been crying my way"--Qualitative analysis of a group of female patients' consultation experiences**. *Family Practice* 1996, **13**(6):498-503.

77. Johansson EE, Hamberg K, Lindgren G, Westman G: **"How could I even think of a job?"--Ambiguities in working life in a group of female patients with undefined musculoskeletal pain**. *Scandinavian Journal of Primary Health Care* 1997, **15**(4):169-174.

78. Johansson EE, Hamberg K, Westman G, Lindgren G: **The meanings of pain: an exploration of women's descriptions of symptoms**. *Social Science & Medicine* 1999, **48**(12):1791-1802.

79. Johsson K, Hedelin H: **Chronic abacterial prostatis: living wiht a troublesome disease affecting many aspects of life**. *Scandinavian Journal of Urology and Nephrology* 2008, **42**:545-550.

80. Jones G, Jenkinson C, Kennedy S: **The impact of endometriosis upon quality of life: a qualitative analysis**. *Journal of Psychosomatic Obstetric Gynecololgy* 2004, **25**:123–133.

81. Keen S, Dowell AC, Hurst K, Klaber JAJ, Tovey P, Williams R: **Individuals with low back pain: how do they view physical activity?** *Family Practice* 1999, **16**(1):39-45.

82. Kelley P, Clifford P: **Coping with chronic pain: assessing narrative approaches**. *Social Work* 1997, **42**(3):266-277.

83. Kugelmann R: **Complaining about chronic pain**. *Social Science & Medicine* 1999, **49**(12):1663-1676.

84. Lachapelle DL, Lavoie S, Boudreau A: **The meaning and process of pain acceptance. Perceptions of women living with arthritis and fibromyalgia**. *Pain Research & Management* 2008, **13**(3):201-210.

85. Laerum E, Indahl A, Sture J: **What is 'the good back-consultation'? A combined qualitative and quantitative study of chronic low back pain patients' interaction with and perceptions of consultations with specialists**. *Journal of Rehabilitation Medicine* 2006, **38**(4):255-262.

86. Lambert BL, Butin DN, Sean DM, Zhao Z, Carr BC, Chen C, Kizis FJ: **Arthritis care: comparison of physicians’ and patients’ views. Semin Arthritis Rheum**. *Seminars in Arthritis and Rheumatism* 2000, **30**(2):100-110.

87. Layzell M: **Back pain management: a patient satisfaction study of services**. *British Journal of Nursing* 2001, **10**(12):800-807.

88. Lempp HK, Hatch SL, Carville SF, Choy EH: **Patients' experiences of living with and receiving treatment for fibromyalgia syndrome: a qualitative study**. *BMC Musculoskeletal Disorders* 2009, **10**.

89. Liddle SD, Baxter GD, Gracey JH: **Chronic low back pain: patients' experiences, opinions and expectations for clinical management**. *Disability & Rehabilitation* 2007, **29**(24):1899-1909.

90. Liedberg GM, Henriksson CM: **Factors of importance for work disability in women with fibromyalgia: an interview study**. *Arthritis & Rheumatism: Arthritis Care & Research* 2002, **47**(3):266-275.

91. Lillrank A: **Back pain and the resolution of diagnostic uncertainty in illness narratives**. *Social Science & Medicine* 2003, **57**(6):1045-1054.

92. Lofgren M, Ekholm J, Ohman A: **'A constant struggle': successful strategies of women in work despite fibromyalgia**. *Disability and Rehabilitation* 2006, **28**(7):447-455.

93. Lundberg M, Styf J, Bullington J: **Experiences of moving with persistent pain--a qualitative study from a patient perspective**. *Physiotherapy Theory & Practice* 2007, **23**(4):199-209.

94. Madden S, Sim J: **Creating meaning in fibromyalgia syndrome**. *Social Science and Medicine* 2006, **63**(11):2962-2973.

95. Magnussen L, Nilsen S, Raheim M: **Barriers against returning to work--as perceived by disability pensioners with back pain: a focus group based qualitative study**. *Disability & Rehabilitation* 2007, **29**(3):191-197.

96. Manderson L, Warren N, Markovic M: **Circuit Breaking: Pathways of Treatment Seeking for Women With Endometriosis in Australia**. *Qualitative Health Research* 2008, **18**:522-534.

97. Mannerkorpi K, Kroksmark T, Ekdahl C: **How patients with fibromyalgia experience their symptoms in everyday life**. *Physiotherapy Research International* 1999, **4**(2):110-122.

98. Markovic M, Manderson L, Warren N: **Endurance and contest: women's narratives of endometriosis**. *Health* 2008, **12**:349-367.

99. Mason B: **A relational approach to the management of chronic pain**. *Clinical Psychology* 2004, **35**:17-20.

100. May CR, Rose MJ, Johnstone FC: **Dealing with doubt. How patients account for non-specific chronic low back pain**. *Journal of Psychosomatic Research* 2000, **49**(4):223-225.

101. May SJ: **Patient satisfaction with management of back pain. Part 1: what is satisfaction? Review of satisfaction with medical management**. *Physiotherapy (London)* 2001, **87**(1):4-9.

102. May S: **Patients' attitudes and beliefs about back pain and its management after physiotherapy for low back pain**. *Physiotherapy Research International* 2007, **12**(3):126-135.

103. McGowan L, Luker K, Creed F, Chew-Graham CA: **How do you explain a pain that can't be seen?: the narratives of women with chronic pelvic pain and their disengagement with the diagnostic cycle**. *British Journal of Health Psychology* 2007, **12**(Pt 2):261-274.

104. McIntosh A, Shaw CF: **Barriers to patient information provision in primary care: patients' and general practitioners' experiences and expectations of information for low back pain**. *Health Expectations* 2003, **6**(1):19-29.

105. McPhillips-Tangum CA, Cherkin DC, Rhodes LA, Markham C: **Reasons for repeated medical visits among patients with chronic back pain**. *Journal of General Internal Medicine* 1998, **13**(5):289-295.

106. Mengshoel AM, Heggen K: **Recovery from fibromyalgia - previous patients' own experiences**. *Disability and Rehabilitation* 2004, **26**(1):46-53.

107. Moore J, Ziebland S, Kennedy S: **‘People sometimes react funny if they’re not told enough’: women’s views about the risks of diagnostic laparoscopy**. *Health Expectations* 2002, **5**:302-309.

108. Morris AL: **Patients' perspectives on self-management following a back rehabilitation programme**. *Musculoskeletal Care* 2004, **2**(3):165-179.

109. Ong BN, Hooper H: **Involving users in low back pain research**. *Health Expectations* 2003, **6**(4):332-341.

110. Ong BN, Hooper H: **Comparing clinical and lay accounts of the diagnosis and treatment of back pain**. *Sociology of Health & Illness* 2006, **28**(2):203-222.

111. Ong BN, Hooper H, Dunn K, Croft P: **Establishing self meaning in low back pain narratives**. *Sociological Review* 2004, **52**:532-549.

112. Ong BN, Konstantinou K, Corbett M, Hay E: **Patients' own accounts of sciatica: a qualitative study**. *Spine* 2011, **36**(15):1251-1256.

113. Osborn M, Smith A: **The personal experience of chronic benign lower back pain: An interpretative phenomenological analysis**. *British Journal of Health Psychology* 1998, **3**(Part 1):65-83.

114. Osborn M, Smith JA: **Living with a body separate from the self. The experience of the body in chronic benign low back pain: an interpretative phenomenological analysis**. *Scandinavian Journal of Caring Sciences* 2006, **20**(2):216-222.

115. Osborn M, Smith A: **The fearfulness of chronic pain and the centrality of the therapeutic relationship in containing it: An interpretative phenomenological analysis**. *Qualitative Research in Psychology* 2008, **5**(4):276-288.

116. Patel S, Greasley K, Watson PJ: **Barriers to rehabilitation and return to work for unemployed chronic pain patients: a qualitative study**. *European Journal of Pain: Ejp* 2007, **11**(8):831-840.

117. Paulson M, Danielson E, Larsson K, Norberg A: **Men's descriptions of their experience of nonmalignant pain of fibromyalgia type**. *Scandinavian Journal of Caring Sciences* 2001, **15**(1):54-59.

118. Paulson M, Danielson E, Soderberg S: **Struggling for a tolerable existence: the meaning of men's lived experiences of living with pain of fibromyalgia type**. *Qual Health Res* 2002a, **12**(2):238-249.

119. Paulson M, Norberg A, Danielson E: **Men living with fibromyalgia-type pain: experiences as patients in the Swedish health care system**. *Journal of Advanced Nursing* 2002b, **40**(1):87-95.

120. Price J, Farmer G, Harris J, Hope T, Kennedy S, Mayou R: **Attitudes of women with chronic pelvic pain to the gynaecological consultation: a qualitative study**. *BJOG: An International Journal of Obstetrics & Gynaecology* 2006, **113**(4):446-452.

121. Raak R, Wahren LK: **Health experiences and employment status in subjects with chronic back pain: a long-term perspective**. *Pain Management Nursing* 2006, **7**(2):64-70.

122. Raheim M, Haland W: **Lived Experience of Chronic Pain and Fibromyalgia: Women's Stories From Daily Life**. *Qual Health Res* 2006, **16**(6):741-761.

123. Raymond MC, Brown JB: **Experience of fibromyalgia. Qualitative study**. *Canadian Family Physician* 2000, **46**:1100-1106.

124. Reid M: **An assessment of health needs of chronic low back pain patients from general practice**. *Journal of Health Psychology* 2004, **9**(3):451-462.

125. Rhodes L, McPhillips-Tangum A, Markham C, Klenk R: **The power of the visible: the meaning of diagnostic tests in chronic back pain**. *Social Science and Medicine* 1999, **48**:1189-1203.

126. Rogers WA: **Beneficence in general practice: an empirical investigation**. *Journal of Medical Ethics* 1999, **25**:388-393.

127. Ross MM, Carswell A, Hing M, Hollingworth G, Dalziel WB: **Seniors' decision making about pain management**. *Journal of Advanced Nursing* 2001, **35**(3):442-451.

128. Sallinen M, Kukkurainen ML, Peltokallio L, Mikkelsson M: **Women's narratives on experiences of work ability and functioning in fibromyalgia**. *Musculoskeletal Care* 2010, **8**(1):18-26.

129. Sallinen M, Kukkurainen ML, Peltokallio L: **Finally heard, believed and accepted - Peer support in the narratives of women with fibromyalgia**. *Patient Education and Counseling* 2011, **85**(2):e126-e130.

130. Sanders C, Donovan J, Dieppe P: **The significance and consequences of having painful and disabled joints in older age: co-existing accounts of normal and disrupted biographies**. *Sociology of Health & Illness* 2002, **24**(2):227-254.

131. Satink T, Winding K, Jonsson H: **Daily Occupations With or Without Pain: Dilemmas in Occupational Performance**. *OTJR: Occupation, Participation and Health* 2004, **24**(4):144-150.

132. Savidge CJ, Slade P, Stewart P, Li TC: **Women's Perspectives on their Experiences of Chronic Pelvic Pain and Medical Care**

*Journal of Health Psychology* 1998, **3**:103-116.

133. Schaefer KM: **Struggling to maintain balance: a study of women living with fibromyalgia**. *Journal of Advanced Nursing* 1995, **21**(1):95-102.

134. Schaefer KM: **Health patterns of women with fibromyalgia**. *Journal of Advanced Nursing* 1997, **26**(3):565-572.

135. Schaefer KM: **The Lived Experience of Fibromyalgia in African American Women**. *Holistic Nursing Practice* 2005, **19**(1):17-25.

136. Schers H, Wensing M, Huijsmans Z, vanTulder M, Grol R: **Implementation barriers for general practice guidelines on low back pain a qualitative study**. *Spine* 2001, **26**:E348-353.

137. Seear K: **The etiquette of endometriosis: stigmatisation, menstrual concealment and the diagnostic delay**. *Social Science & Medicine* 2009, **69**(8):1220-1227.

138. Seear K: **The third shift: health, work and expertise among women with endometriosis.** *Health Sociology Review* 2009b, **2**:194-206.

139. Seear K: **‘Nobody really knows what it is or how to treat it’: why women with endometriosis do not comply with healthcare advice. Health Risk Soc 2009c**. *Health Risk and Society* 2009c, **4**:367-385.

140. Seers K, Friedli K: **The patients’ experiences of their chronic non-malignant pain**. *Journal of Advanced Nursing* 1996, **24**(6):1160-1168.

141. Skelton A: **The hidden curriculum of patient education for low back pain in general practice**. *Sociology of Health and Illness* 1998, **20**(1):96-111.

142. Skelton AM, Murphy EA, Murphy RJL, O'Dowd TC: **Patient education for low back pain in general practice**. *Patient Education & Counseling* 1995, **25**:329–334.

143. Skelton AM, Murphy EA, Murphy RJL, O'Dowd T: **Patient's views of low back pain**. *British Journal of General Practice* 1996, **46**:153-156.

144. Skuladottir H, Halldorsdottir S: **The quest for well-being: self-identified needs of women in chronic pain**. *Scandinavian Journal of Caring Sciences* 2011, **25**(1):81-91.

145. Slade SC, Molloy E, Keating JL: **'Listen to me, tell me': a qualitative study of partnership in care for people with non-specific chronic low back pain**. *Clinical Rehabilitation* 2009a, **23**(3):270-280.

146. Slade SC, Molloy E, Keating JL: **People with non-specific chronic low back pain who have participated in exercise programs have preferences about exercise: a qualitative study**. *Australian Journal of Physiotherapy* 2009b, **55**(2):115-121.

147. Slade SC, Molloy E, Keating JL: **Stigma experienced by people with nonspecific chronic low back pain: a qualitative study**. *Pain Medicine* 2009c, **10**(1):143-154.

148. Sloots M, Dekker JHM, Pont M, Bartels EA, Geertzen JHB, Dekker J: **Reasons for drop-out from rehabilitation in patients of Turkish and Moroccan origin with chronic low back pain in The Netherlands: a qualitative**. *Journal of Rehabilitation Medicine* 2010, **42**(6):566-574.

149. Smith JA, Osborn M: **Pain as an assault on the self: an interpretative phenomenological analysis of the psychological impact of chronic benign low back pain**. *Psychology & Health* 2007, **22**(5):517-535.

150. Snelgrove S, Liossi C: **An interpretative phenomenological analysis of living with chronic low back pain**. *British Journal of Health Psychology* 2009, **14**(Pt 4):735-749.

151. Snelgrove S, Edwards S, Liossi C: **A longitudinal study of patients’ experiences of chronic low back pain using interpretative phenomenological analysis: Changes and consistencies**. *Psychology & Health* 2013, **28**(2):121–138.

152. Soderberg S, Norburg A: **Metaphorical pain language among fibromyalgia patients**. *Scandinavian Journal of Caring Sciences* 1995, **9**(1):55-59.

153. Soderberg S, Lundman B, Norberg A: **Struggling for dignity: the meaning of women's experiences of living with fibromyalgia**. *Qualitative Health Research* 1999, **9**(5):575-587.

154. Soderberg S, Lundman B: **Transitions experienced by women with fibromyalgia**. *Health Care Women Int* 2001, **22**(7):617-631.

155. Soderberg S, Lundman B, Norberg A: **The meaning of fatigue and tiredness as narrated by women with fibromyalgia and healthy women**. *Journal of Clinical Nursing* 2002, **11**(2):247-255.

156. Sofaer-Bennett B, Holloway I, Moore A, Lamberty J, Thorp T, O'Dwyer J: **Perseverance by older people in their management of chronic pain: a qualitative study**. *Pain Medicine* 2007, **8**(3):271-280.

157. Sokunbi O, Cross V, Watt P, Moore A: **Experiences of individuals with chronic low back pain during and after their participation in a spinal stabilisation exercise programme -- a pilot qualitative study**. *Manual Therapy* 2010, **15**(2):179-185.

158. Steen E, Haugli L: **From pain to self-awareness--a qualitative analysis of the significance of group participation for persons with chronic musculoskeletal pain**. *Patient Education & Counseling* 2001, **42**(1):35-46.

159. Strong J, Ashton R, Chant D, Cramond T: **An investigation of the dimensions of chronic low back pain: the patients' perspectives**. *British Journal of Occupational Therapy* 1994, **57**(6):204-209.

160. Strong J, Large RG: **Coping with chronic low back pain: an idiographic exploration through focus groups**. *International Journal of Psychiatry in Medicine* 1995, **25**(4):371-387.

161. Strunin L, Boden LI: **Family consequences of chronic back pain**. *Social Science & Medicine* 2004, **58**(7):1385-1394.

162. Strzempko F, Chesla C: **Relational patterns of couples living with chronic pelvic pain from endometriosis**. *Qualitative Health Research* 2007, **17**(5):571-585.

163. Sturge-Jacobs M: **The experience of living with fibromyalgia: confronting an invisible disability**. *Research & Theory for Nursing Practice* 2002, **16**(1):19-31.

164. Tarasuk T, Eakin J: **The problem of legitimacy in the experience of workrelated back injury**. *Qualitative Health Research* 1995, **5**(2):204-221.

165. Tavafian SS, Gregory D, Montazeri A: **The experience of low back pain in Iranian women: a focus group study**. *Health Care for Women International* 2008, **29**(4):339-348.

166. Teh CF, Karp JF, Kleinman A, Reynolds C, Weiner DK, Cleary P: **Older people's experiences of patient-centred treatment for chronic pain: a qualitative study**. *Pain Medicine* 2009, **10**(3):521-530.

167. Thomas S: **A Phenomenological study of Chronic Pain**. *Western Journal of Nursing Research* 2000, **22**(6):683-705.

168. Thorne S, McGuinness L, Con A, Cunningham M, Harris SR: **Health care communication issues in fibromyalgia: an interpretive description**. *Physiotherapy Canada* 2004, **56**(1):31-38.

169. Toye F, Barker K: **'Could I be imagining this?' - the dialectic struggles of people with persistent unexplained back pain**. *Disability & Rehabilitation* 2010, **32**(21):1722-1732.

170. Toye F, Barker K: **Persistent non-specific low back pain and patients’ experience of general practice: a qualitative study**. *Primary Health Care Research & Development* 2012a, **13**(72-84).

171. Toye F, Barker K: **‘I can’t see any reason for stopping doing anything, but I might have to do it differently’ – restoring hope to patients with persistent non-specific low back pain – a qualitative study**. *Disability & Rehabilitation* 2012b, **34**(11):894-903.

172. Tveito TH, Shaw WS, Huang YH, Nicholas M, Wagner G: **Managing pain in the workplace: a focus group study of challenges, strategies and what matters most to workers with low back pain**. *Disability & Rehabilitation* 2010, **32**(24):2035-2045.

173. Undeland M, Malterud K: **The fibromyalgia diagnosis: hardly helpful for the patients? A qualitative focus group study**. *Scandinavian Journal of Primary Health Care* 2007, **25**(4):250-255.

174. Underwood MR, Harding G, Klaber J, Uk UKBEAMt: **Patient perceptions of physical therapy within a trial for back pain treatments (UK BEAM) [ISRCTN32683578]**. *Rheumatology* 2006, **45**(6):751-756.

175. Vroman K, Warner R, Chamberlain K: **Now let me tell you in my own words: narratives of acute and chronic low back pain**. *Disability & Rehabilitation* 2009, **31**(12):976-987.

176. Wade BL, Shantall HM: **The meaning of chronic pain: a phenomenological analysis**. *South African Journal of Physiotherapy* 2003, **59**(1):10-19.

177. Walker J, Holloway I, Sofaer B: **In the system: the lived experience of chronic back pain from the perspectives of those seeking help from pain clinics**. *Pain* 1999, **80**(3):621-628.

178. Walker J, Sofaer B, Holloway I: **The experience of chronic back pain: accounts of loss in those seeking help from pain clinics**. *European Journal of Pain: Ejp* 2006, **10**(3):199-207.

179. Warwick R, Joseph S, Cordle C, Ashworth P: **Social support for women with chronic pelvic pain: what is helpful from whom?** *Psychology & Health* 2004, **19**(1):117-134.

180. Werner A, Steihaug S, Malterud K: **Encountering the continuing challenges for women with chronic pain: recovery through recognition**. *Qualitative Health Research* 2003a, **13**(4):491-509.

181. Werner A, Malterud K: **It is hard work behaving as a credible patient: encounters between women with chronic pain and their doctors**. *Social Science & Medicine* 2003b, **57**(8):1409-1419.

182. Werner A, Isaksen LW, Malterud K: **'I am not the kind of woman who complains of everything': illness stories on self and shame in women with chronic pain**. *Social Science & Medicine* 2004, **59**(5):1035-1045.

183. Whelan E: **‘No one agrees except for those of us who have it’: endometriosis patients as an epistemological community**. *Sociology of Health & Illness* 2007, **29**(7):957-982.

184. White S, Siebold C: **Walk a mile in my shoes: an auto-ethnographic study**. *Contemporary Nurse* 2008, **30**(1):57-68.

185. Whitney ML: **Importance of lay organisations for coping with endometriosis**. *Medicine* 1998, **43**:331-334.

186. Young AE, Wasiak R, Phillips L, Gross DP: **Workers' perspectives on low back pain recurrence: "it comes and goes and comes and goes, but it's always there"**. *Pain* 2011, **152**(1):204-211.

187. Zadinsky JK, Boyle JS: **Experiences of women with chronic pelvic pain**. *Health Care for Women International* 1996, **17**(3):223-232.
